# The Association of Kidney Function and Cardiovascular Disease Indices with Pro-Oxidant and Antioxidant Markers in CKD

**DOI:** 10.3390/antiox15101340

**Published:** 2026-10-16

**Authors:** Ariti Tsinari, Stefanos Roumeliotis, Ioannis Eleftherios Neofytou, Athanasios Roumeliotis, Garyfallia Varouktsi, Aikaterini Stamou, Ioannis Kontogiorgos, Konstantinos Leivaditis, Andrej Veljkovic, Gordana Kocic, Vassilios Liakopoulos

**Affiliations:** 1 Second Department of Nephrology, School of Medicine, AHEPA Hospital, Aristotle University of Thessaloniki, 1 St. Kyriakidi Street, 54636 Thessaloniki, Greecejohn_neofytou_@hotmail.com (I.E.N.);; 2 Faculty of Medicine, University of Nis, 18108 Nis, Serbia; veljkovicandrej@yahoo.com; 3 Faculty of Pharmacy, University of Novi Sad, 21102 Novi Sad, Serbia; kocicrg@yahoo.co.uk; 4 Serbian Academy of Sciences and Arts, 11000 Belgrade, Serbia

**Keywords:** advanced oxidation products, arterial stiffness, cardiovascular disease, carotid intima media thickness, catalase, chronic kidney disease, malondialdehyde, oxidized albumin, superoxide dismutase, xanthine oxidase

## Abstract

Oxidative stress (OS) is highly prevalent in chronic kidney disease (CKD), yet its clinical significance and association with cardiovascular disease (CVD) has not been yet clearly demonstrated. In this cohort study, 477 adults with CKD at various stages and 20 healthy controls were enrolled. In all participants, an extensive panel of OS biomarkers was measured. Malondialdehyde (MDA) was correlated with glomerular filtration rate (eGFR) and carotid intima media thickness (cIMT) (*p* = 0.008 and *p* = 0.024, respectively, according to Spearman’s rho). Advanced oxidation protein products (AOPPs) were correlated with the urine protein-to-creatinine ratio (uPCR), parathormone (PTH) and eGFR (*p* = 0.048, *p* = 0.025 and *p* = 0.045 respectively, according to Spearman’s rho) and oxidized albumin (oxALB) was correlated with PTH (*p* = 0.032) and pulse wave velocity (PWV) (*p* = 0.017). The antioxidant enzyme superoxide dismutase (SOD) was correlated with PWV (*p* = 0.042), urea (*p* < 0.001), uPCR (*p* = 0.024), and eGFR (*p* < 0.001). Meanwhile, catalase (CAT) was correlated with eGFR (*p* = 0.029) and indices of central pressure. Xanthine oxidase (XO) was correlated with the duration of CVD and PWV. Multivariate analysis showed that kidney function and/or arterial stiffness and CVD were independent predictors of all OS markers.

## 1. Introduction

Oxidative stress (OS) is a phenomenon caused by an imbalance between production/accumulation of reactive oxygen species (ROS) in cells and tissues and the ability of a biological system to detoxify these reactive products in favor of the former [1]. OS has deleterious effects on a wide range of biomolecules and causes severe structural and functional damage to lipids, enzymes, proteins and nucleic acids. Lipoprotein particles and phospholipids in cell membranes undergo lipid peroxidation, giving rise to highly reactive and toxic molecules like malondialdehyde (MDA). Oxidative damage to proteins leads to modifications of amino acids and the generation of advanced oxidation protein products (AOPPs) and, particularly, oxidized forms of human albumin (oxALB) [2]. The imbalance between pro- and anti-oxidant enzymes is reflected in enzymatic activity with predominance of radical-producing isoforms such as xanthine oxidase (XO), while the activity of endogenous antioxidant enzymes such as superoxide dismutase (SOD) and catalase (CAT)—which under physiological conditions safeguard cell function—is diminished [3].

There is growing evidence that OS plays a pivotal role in the onset and progression of various human diseases, including cardiovascular diseases (CVD) and chronic kidney disease (CKD), which are degenerative diseases of accelerated cellular aging [4]. However, existing data are derived mainly from experimental studies. In clinical settings, OS has been studied in several CKD cohorts [5,6,7,8,9,10].

In CKD, OS occurs in early stages, is gradually aggravated in parallel with the deterioration of kidney function and is further exacerbated in the end stage due to the dialysis procedure. Hemodialysis (HD) might trigger OS in a greater degree than peritoneal dialysis (PD) due to the bioincompatibility of this method. Although accumulating clinical and experimental evidence from various CKD populations suggests that OS may be associated with adverse clinical outcomes, including decline in kidney function, atherosclerosis, cardiovascular events, and mortality [11,12,13,14,15], there are currently no clear guidelines or clinical recommendations supporting the routine assessment of OS in everyday clinical practice or the use of antioxidant supplementation in patients with CKD. Therefore, despite the substantial body of evidence derived from clinical and experimental studies, OS has not yet gained the clinical attention that its potential role in CKD might warrant. This may be partly attributed to the heterogeneity of the available studies, including the relatively small sample sizes, the inclusion of specific CKD populations or stages—particularly patients receiving hemodialysis—and the assessment of only one or two OS markers in many studies [16,17,18,19,20]. Such an approach may provide only a limited view of the complex interplay between OS, kidney dysfunction, and vascular injury and may therefore limit our understanding of the overall relationship between OS and cardiovascular complications in CKD.

To address the gaps in the clinical characterization of OS in CKD—including the need for large and representative cohorts, a comprehensive assessment using multiple OS biomarkers, integrated evaluation of vascular dysfunction and damage, and prospective clinical follow-up—we designed a large, multicenter clinical study with a baseline cross-sectional assessment and a prospective follow-up period. In a large cohort of patients at different CKD stages, we assessed OS through multiple molecular indices and recorded medical history, CVD status, traditional and non-traditional risk factors, as well as markers of vascular calcification such as carotid atherosclerosis and arterial stiffness. By combining biochemical, clinical and vascular information in a single dataset, we aimed to provide a holistic view of the redox milieu in CKD and to clarify how disturbances in OS are linked to cardiovascular endpoints and CKD progression.

## 2. Materials and Methods

### 2.1. Ethical Considerations

The study was conducted according to the principles of the Declaration of Helsinki and the International Council for Harmonization Good Clinical Practice (GCP) guideline E6. The protocol was reviewed and approved by the Ethics Committee of the Medical School of Aristotle University of Thessaloniki (approval no. 139, 14 March 2024). Prior to enrollment, all participants received detailed oral and written information regarding the non-interventional nature of the study and its objectives and provided written informed consent. Personal identifiers were removed from the analytical dataset; sensitive information was stored in anonymous form, and each participant was assigned a study code for analysis (pseudonymized data).

### 2.2. Study Design and Population

**Figure 1:**
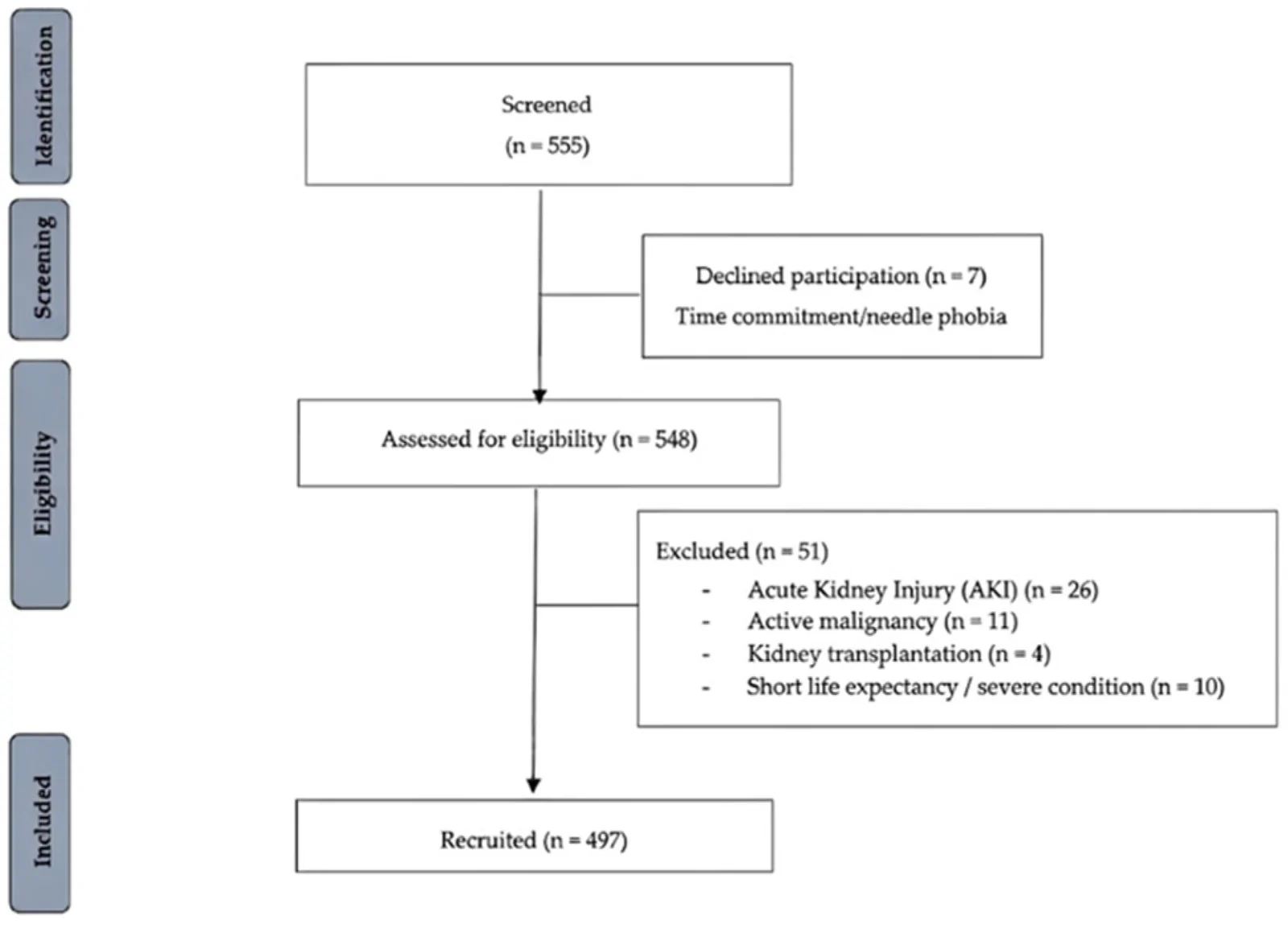
Enrollment flowchart.

This multi-center, observational cohort study included a cross-sectional baseline assessment and a prospective part. The study was strictly non-interventional; all diagnostic and therapeutic procedures were performed as part of routine clinical care, and study activities were limited to clinical examination, structured data collection, vascular assessments, and blood and urine sampling. The present analysis is cross-sectional and is based on clinical and biochemical data collected at baseline. Figure 1 shows the enrolment flowchart. Following enrollment, participants were prospectively followed for up to 24 months for predefined clinical outcomes. The primary outcome was all-cause mortality, while secondary outcomes included major adverse cardiovascular events (MACE), comprising fatal and non-fatal cardiovascular events, and longitudinal decline in kidney function. All-cause mortality and cardiovascular events were recorded prospectively throughout the follow-up period. At the end of the 24-month follow-up, kidney function was reassessed using eGFR and albuminuria to evaluate longitudinal changes in kidney function.

A total of 497 subjects were included: 477 patients with various stages of CKD (45 patients with stages 1 and 2, 78 patients with stage 3, 60 patients with stage 4, 294 patients with stage 5 on renal replacement therapy (RRT), including 164 on HD and 130 on PD, and 20 healthy age- and gender-matched controls. Healthy participants were defined as volunteers with normal renal function and no proteinuria who were currently not taking medication for any acute or chronic illness. The inclusion criteria were age > 18 years old and a diagnosis of CKD based on the Kidney Disease: Improving Global Outcomes (KDIGO) 2012 criteria [21]. Exclusion criteria included acute illness or an active severe inflammatory condition; hospitalization for any cause within the 3 months prior to enrollment; acute kidney injury (AKI); anticipated renal transplantation within the next 4 months; pregnancy or breastfeeding; a history of drug or alcohol abuse; active malignancy or ongoing oncologic treatment; and any medical condition significantly limiting life expectancy (<1 year). Patients who declined to provide informed consent were also excluded.

After enrollment, all patients were to be followed for a planned median of 24 months with end points of mortality, CVD events and kidney function decline.

### 2.3. Clinical and Medical History Data

Baseline clinical and demographic information was obtained for all participants through structured interviews, measurements and review of electronic medical records. The following variables were recorded:

- Demographics and kidney disease characteristics: age, sex, CKD stage (according to the KDIGO 2012 criteria), urea, serum creatinine, urine albumin and protein-to-creatinine ratio (uACR and uPCR) and primary cause of nephropathy; for patients on RRT, modality (HD or PD), total duration of dialysis, and peritoneal transport status where applicable. To determine peritoneal transport status, a 4 h peritoneal equilibration (PET) test was performed in PD patients following the guidelines of the International Society for Peritoneal Dialysis (ISPD). In HD patients, Kt/V was measured to assess dialysis adequacy. This procedure has been described in detail elsewhere [22].
- Comorbidities: presence and duration of hypertension, diabetes mellitus, dyslipidemia, and established CVD, further categorized into angina, myocardial infarction (MI), heart failure (clinical and/or imaging-based diagnosis), stroke/transient ischemic attack, and peripheral artery disease (PAD). CVD history was strictly defined based on officially documented and clinically validated events in the patients’ medical records. Although the high prevalence of major CVD risk factors—such as diabetes mellitus and arterial hypertension—in our cohort suggests that the true burden of CVD is very high, we deliberately excluded unverified or suspected cases to maintain methodological rigor and ensure the reliability of our data.
- Current medication.

### 2.4. Anthropometric Measurements

Baseline anthropometric measurements were obtained during a morning visit (08:00–10:00), after an overnight fast (at least 8 h), to minimize variation in hydration status and therefore in body weight. Body weight (BW, kg) was measured using a calibrated electronic scale while the participants were wearing light clothing and standing in the center of the platform for even weight distribution. For HD patients, post-dialysis body weight was recorded after the mid-week treatment session, while for PD patients, body weight was recorded after complete drainage of the peritoneal cavity to reduce the influence of ultrafiltration and intraperitoneal fluid on BW.

Standing height (BH, cm) was measured using a validated stadiometer, with participants standing barefoot, scapulae and heels in contact, and the head positioned in the Frankfurt horizontal plane. Body mass index (BMI, kg/m^2^) was calculated using the Adolphe Quetelet formula: BMI = BW/BH^2^.

### 2.5. Standard Laboratory Assessments

Non-dialysis patients attended the nephrology outpatient clinic in the morning (08:00–10:00) after an overnight fast of at least 8 h. Venous blood samples were drawn by standard venipuncture and processed within 2 h at the hospital’s microbiology laboratory using validated automated methods. For HD patients, blood sampling was performed during the mid-week HD session, immediately before dialysis initiation (pre-dialysis) and prior to heparin or saline administration, to capture steady-state pre-dialysis concentrations. For PD patients, blood samples were collected at routine monthly clinic visits in the morning after an overnight fast; sampling was performed after complete drainage of the overnight dialysate and before the first daytime exchange.

A complete blood count, including red cell distribution width, was obtained using an automated hematology analyzer (Sysmex XE 5000, Kobe, Japan). Standard biochemical parameters were measured, including serum glucose, albumin, sodium, potassium, calcium, hydrogen phosphate, urea, creatinine, liver enzymes (aspartate aminotransferase, alanine aminotransferase), lipid profile [total cholesterol, low-density lipoprotein cholesterol (LDL), high-density lipoprotein cholesterol (HDL), triglycerides (TRG)], intact parathormone (PTH), C-reactive protein (CRP), and glycosylated hemoglobin (HbA1c), using enzymatic colorimetric or immunoturbidimetric assays on automated platforms (Roche Cobas 6000/Roche Cobas c501). First-morning spot urine samples were analyzed for creatinine, albumin and total protein (Roche Cobas c501), and uACR and uPCR were calculated from morning urine samples. Estimated glomerular filtration rate (eGFR) was calculated using the 2009 CKD-EPI creatinine equation [21].

### 2.6. Oxidative Stress Biomarker Determination

For OS biomarker assessment, samples were centrifuged, and the separated serum or plasma aliquots were stored at −80 °C until analysis. Then, batched samples were transported on dry ice to the Institute of Biochemistry, Faculty of Medicine, University of Niš, Serbia, under continuous temperature control and analyzed in a certified redox research laboratory. Quality control samples were included in each run, with efforts to maintain intra- and inter-assay coefficients of variation below 10%.

The following OS biomarkers were determined:

#### 2.6.1. Markers of Oxidative Damage

MDA: Lipid peroxidation was assessed by measuring plasma MDA using a thiobarbituric acid reactive substances (TBARS) spectrophotometric assay based on the formation of a colored MDA–thiobarbituric acid adduct [23]; results were expressed in μmol/L.

AOPPs: AOPPs were measured spectrophotometrically at 340 nm, based on the formation of a stable yellow-colored product by adding acetic acid and potassium iodide solution to the plasma sample [24]. The result was expressed as mmol/L chloramine-T equivalents.

OxALB: Oxidatively modified human serum albumin was quantified spectrophotometrically at 470 nm based on the measurement of the cobalt-binding sites on the N-terminal end of human blood albumin through determination of free-unbound cobalt [25].

#### 2.6.2. Pro-Oxidant Enzymes

XO activity: XO, the pro-oxidant form of xanthine oxidoreductase, was determined indirectly by measuring the rate of uric acid formation during the oxidation of xanthine. Enzymatic activity was assessed spectrophotometrically and expressed in mU/L [26].

#### 2.6.3. Antioxidant Enzymes

CAT activity: CAT activity, reflecting enzymatic degradation of hydrogen peroxide, was measured by a spectrophotometric assay in which residual hydrogen peroxide forms a stable yellow complex with ammonium molybdate at 405 nm as described by Goth [27]; activity was expressed in cat/L.

SOD activity: SOD activity was determined using a pyrogallol autoxidation assay in the presence of released hydrogen peroxide (H_2_O_2_). SOD inhibits the formation of the colored pyrogallol oxidation product, pyrogallol-ortho-quinone [28]; activity was expressed in U/mL.

### 2.7. Arterial Stiffness and Hemodynamic Measurements

Twenty-four-hour ambulatory blood pressure monitoring (ABPM) and pulse wave analysis were performed using the Mobil-O-Graph device (IEM, Stolberg, Germany), a clinically validated oscillometric system that records brachial blood pressure (BP) and reconstructs central arterial waveforms. The device first measures brachial systolic and diastolic BP, then reinflates the cuff at a lower pressure to capture pulse waveforms, from which central hemodynamic parameters are derived using the ARCSolver algorithm. The following peripheral and central parameters were obtained: peripheral systolic BP (SBP), diastolic BP (DBP), mean BP, heart rate (HR), peripheral pulse pressure (pPP), central systolic (cSBP) and diastolic blood pressure (cDBP), central pulse pressure (PP), augmentation index (AIx), and aortic pulse wave velocity (PWV), as an index of arterial stiffness.

### 2.8. Carotid Ultrasound and Intima–Media Thickness

Carotid ultrasound was performed by an experienced clinician using a high-resolution B-mode ultrasound system equipped with a linear 7.5-MHz transducer (Samsung V4 ultrasound, Samsung Medison, Seoul, South Korea). The clinician performing the ultrasound was blinded to the participants’ renal function and CKD group to minimize potential observer and measurement bias. Carotid intima–media thickness (cIMT) was measured at three different sites: on the far wall of the common carotid artery, carotid bulb, and internal carotid artery bilaterally, following standard protocols for subclinical atherosclerosis assessment. cIMT was defined as the distance between the leading echogenic edge of the lumen–intima interface and the leading echogenic edge of the media–adventitia interface. For each of the three arterial segments on both sides, cIMT values were obtained over a 1-cm arterial segment, and mean cIMT was calculated as the average of these measurements, as described before [29].

All examined carotid segments were systematically evaluated for the presence of atherosclerotic plaques, defined as a focal structure encroaching into the arterial lumen with a thickness ≥ 1.5 mm or ≥50% of the surrounding cIMT. Carotid plaque presence was recorded as a marker of atherosclerotic disease.

### 2.9. Statistical Analysis

Data were analyzed using IBM SPSS Statistics version 29 (IBM Corp., Armonk, NY, USA). The distribution of continuous variables was assessed with the Kolmogorov–Smirnov test for normality. Normally distributed variables are presented as mean ± standard deviation, whereas non-normally distributed variables are expressed as median (minimum–maximum). Categorical variables are summarized as counts and percentages. Between-group differences were evaluated using the independent-samples *t*-test or ANOVA for normally distributed data. For non-normally distributed data, the Mann-Whitney U or Kruskal–Wallis tests were applied.

Correlations between continuous variables, particularly between OS biomarkers and clinical or laboratory parameters, were examined using Spearman’s rank correlation coefficient, given the frequent deviation from normality and potential outliers. Given the exploratory nature of the initial bivariate correlations, candidate variables were identified for multivariable modeling based on unadjusted statistical significance. However, to rigorously address the multiple testing problem and control the false positive reporting rate, the Benjamini-Hochberg False Discovery Rate (FDR) procedure was subsequently calculated for the entire correlation matrix [30]. The FDR procedure was selected over the traditional Bonferroni correction to effectively limit false discoveries while preserving statistical power and minimizing Type II errors in this multimorbid cohort. The complete FDR-adjusted significance thresholds for all bivariate correlations are available in Supplementary Table S2.

To investigate independent associations, multiple linear regression models were constructed with OS markers as dependent variables and the variables that were correlated with these markers in previous analyses (e.g., age, sex, CKD stage, dialysis modality, diabetes, BP, lipid profile) as independent variables. Sensitivity analyses were conducted in prespecified subgroups (HD and PD patients), using non-parametric tests (Kruskal–Wallis) and stratified regression models to explore the robustness of observed associations across different RRT modalities and CKD phenotypes. A two-sided *p*-value < 0.05 was considered statistically significant for all tests.

## 3. Results

A total of 497 subjects were included in the study. There were 45 patients with CKD stages 1 + 2 (9.1%), 78 patients with CKD stage 3 (15.7%), 60 patients with CKD stage 4 (12.1%), 164 patients with CKD stage 5 undergoing HD (33%), 130 patients with CKD stage 5 treated with PD (26.2%) and 20 healthy controls (4%) from the general population with similar demographic characteristics. The baseline characteristics are shown in Table 1.

Our cohort showed a heavy CVD burden. The overall prevalence of documented CVD was 49.7%, distributed as follows: 15.4% in CKD stages 1 + 2, 58.4% in stage 3, 57.1% in stage 4, and 53.5% in stage 5. In total, 74.8% of participants were hypertensive, 52.3% were diagnosed with dyslipidemia and 42.3% were diabetic, with a mean duration since diagnosis of 20 years. Of our patients, 19.2% had experienced a MI, 8.5% had had a stroke, 17.6% had reported at least one episode of angina, 28.4% were diagnosed with heart failure and 22.9% were recorded with signs or symptoms of PAD. A carotid atheromatous plaque was found in a significant 63.7% of cases. The median duration of CVD was 10 years. For patients on RRT, 55.7% were treated with HD for an average duration of 16.8 ± 37.5 months and 44.2% were treated with PD for 9.8 ± 23.8 months.

Baseline characteristics of the healthy controls included a median age of 50 years [21,22,23,24,25,26,27,28,29,30,31,32,33,34,35,36,37,38,39,40,41,42,43,44,45,46,47,48,49,50,51,52,53,54,55,56,57,58,59,60,61,62,63,64,65,66,67], and 35% (n = 7) were male; median laboratory values were as follows: eGFR 107 mL/min/1.73 m^2^ [100–140], serum creatinine 0.68 mg/dL [0.57–0.96], serum urea 28 mg/dL [16,17,18,19,20,21,22,23,24,25,26,27,28,29,30,31,32,33], uACR 0.01 g/g [0.03–0.074], and uPCR 0.009 g/g [0.002–0.07].

### 3.1. Biomarkers of Oxidative Stress

#### 3.1.1. MDA

**Figure 2:**
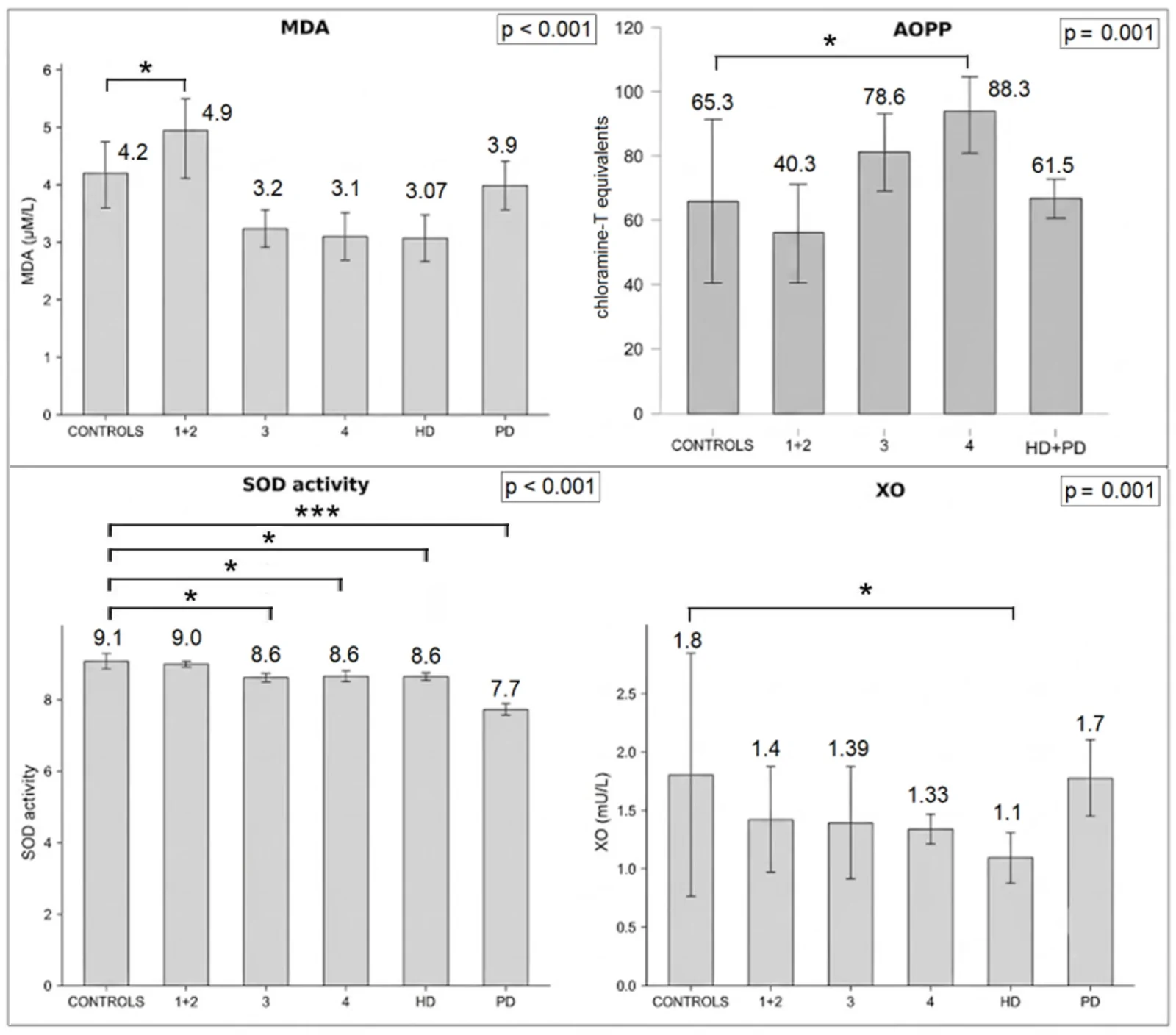
Oxidative stress marker levels in healthy controls compared to specific CKD subgroups. The *p*-values in the upper right corner of each panel indicate the overall statistical significance calculated using the non-parametric Kruskal–Wallis test. Asterisks denote statistically significant pairwise differences between specific chronic kidney disease subgroups and the healthy control group, following adjustment for multiple comparisons via the Bonferroni correction (* *p* < 0.05; *** *p* < 0.001 vs. controls). Abbreviations: AOPP, advanced oxidation protein product; CKD, chronic kidney disease; MDA, malondialdehyde; SOD, superoxide dismutase; XO, xanthine oxidase.

**Figure 3:**
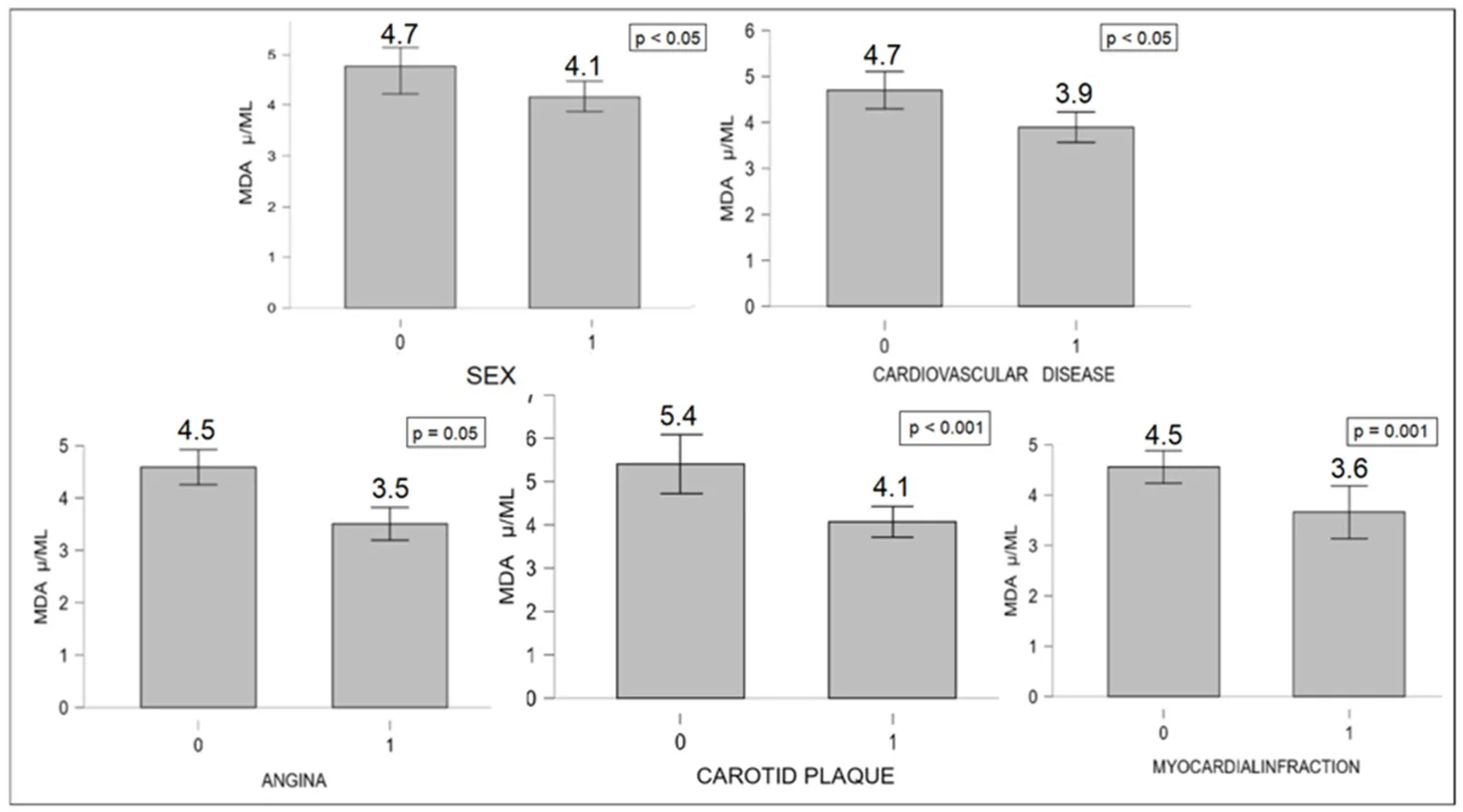
Malondialdehyde levels according to sex, history of cardiovascular conditions, and presence of carotid atheromatous plaque. *p*-values indicate the statistical significance of differences in malondialdehyde (MDA) levels between two independent groups (e.g., presence vs. absence of a condition, or male vs. female). Because the data were non-normally distributed, *p*-values were calculated using the non-parametric Mann–Whitney U test. 0: absence of condition; 1: presence of condition. For sex, 0 = female and 1 = male. Data are presented as median ± SE.

The median value of MDA in the study population was 3.56 μM/L (1.64–25.71). Using Spearman’s rho correlation, levels of MDA correlated significantly with eGFR (r = 0.126, *p* = 0.008), age (r = −0.167, *p* = 0.001), hemoglobin (r = 0.136, *p* = 0.005), potassium (r = 0.171, *p* < 0.001), calcium (r = 0.109, *p* = 0.024), total cholesterol (r = 0.181, *p* < 0.001), LDL (r = 0.161, *p* = 0.001), and IMT (r = −0.122, *p* = 0.024) (Table 2). The Kruskal–Wallis test showed that MDA levels varied significantly across CKD stages (*p* < 0.001) (Figure 2), gender (*p* = 0.041), as well as among patients with and without a history of CVD (*p* = 0.008), MI (*p* = 0.001), angina (*p* = 0.05) and the presence of an atheromatous carotid plaque (*p* < 0.001) (Figure 3). In multiple regression analysis, plaque presence (B = −1.12, 95% CI −1.85 to −0.39, *p* = 0.003) and eGFR (B = 0.013, 95% CI 0.001 to 0.024, *p* = 0.027) emerged as the sole independent predictors of MDA levels, while sex, Hb, calcium, and CVD were also evaluated as independent predictors but were excluded (Table 3).

#### 3.1.2. AOPPs

The median value of AOPPs, expressed as mmol/L chloramine-T equivalents, was 68.18 (0.17–239.43). AOPPs were significantly correlated with PTH (r = −0.109, *p* = 0.025), total cholesterol (r = −0.130, *p* = 0.008), LDL (r = −0.108, *p* = 0.026), uPCR (r = −0.117, *p* = 0.048), and eGFR (r = 0.096, *p* = 0.045), as determined by Spearman’s rho (Table 2). The Kruskal–Wallis test showed that AOPPs levels varied significantly among patients with different stages of CKD (*p* = 0.001) (Figure 2). Multivariate regression analysis revealed that PTH (B = −0.046, 95% CI −0.075 to −0.016, *p* = 0.003) and total cholesterol (B = −0.162, 95% CI −0.280 to −0.045, *p* = 0.007) were independent predictors of AOPPs, whereas eGFR, LDL, and proteinuria were evaluated as independent predictors but were excluded (Table 3).

#### 3.1.3. OxALB

The median value of oxidatively modified albumin was 0.08 (0–0.85) absorbance units, correlating significantly with PWV (r = −0.134, *p* = 0.017) and PTH (r = 0.109, *p* = 0.032) (Table 2). Multivariate regression analysis confirmed that PWV was the sole independent predictor of oxALB (B = −0.009, 95% CI −0.017 to −0.001, *p* = 0.031); PTH was also evaluated as an independent predictor but was excluded (Table 3).

#### 3.1.4. XO

**Figure 4:**
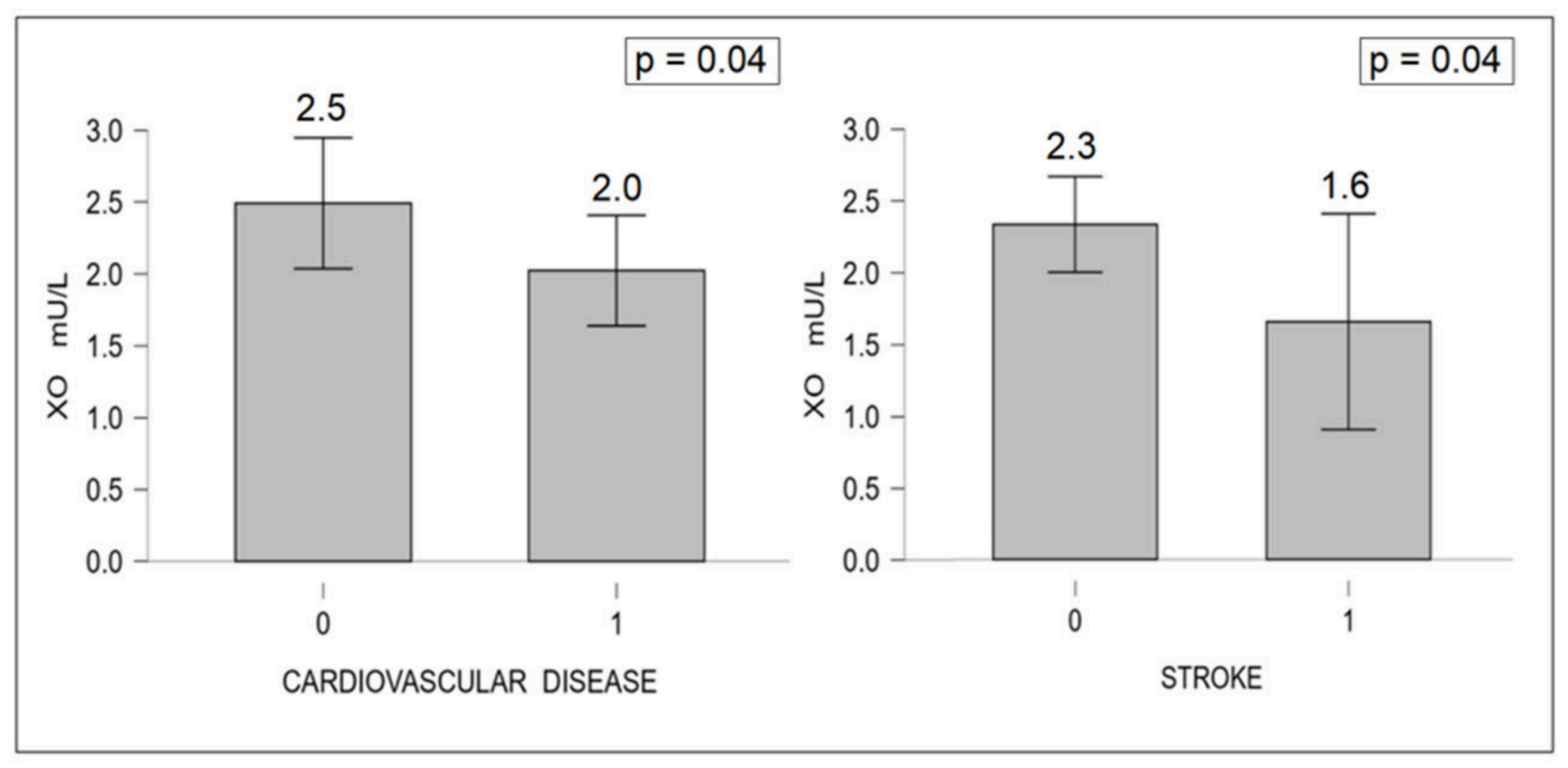
Xanthine oxidase activity in patients with and without a history of cardiovascular disease and stroke. *p*-values indicate the statistical significance of differences in xanthine oxidase (XO) activity between patients with and without a history of cardiovascular disease and stroke, calculated using the non-parametric Mann–Whitney U test. 0: absence of condition; 1: presence of condition. Data are presented as median ± SE.

The median value of XO activity was 1.37 mU/L (0.03–41.28), correlating with PWV and pPP (r = −0.121, *p* = 0.026 and r = −0.117, *p* = 0.032, respectively), age (r = −0.102, *p* = 0.037), and the duration of CVD in years (r = −0.100, *p* = 0.042) (Table 2). Applying the Kruskal–Wallis test, XO activity varied significantly across CKD stage categories (*p* = 0.001) (Figure 2) and among patients with and without a history of CVD (*p* = 0.037) or stroke (*p* = 0.037, Figure 4). Regression analysis showed that PWV was independently associated with XO activity (B = −0.228, 95% CI −0.415 to −0.040, *p* = 0.017) (Table 3), whereas age, CVD, peripheral pulse pressure, and CKD group were evaluated as independent predictors but were excluded (Table 3).

#### 3.1.5. SOD

SOD activity in the study population had a median value of 8.55 U/mL (0.53–9.68). Spearman’s rho analysis showed a significant correlation of SOD with PWV (r = 0.109, *p* = 0.042), hemoglobin (r = 0.110, *p* = 0.023), urea (r = −0.295, *p* < 0.001), sodium (r = 0.217, *p* < 0.001), hydrogen phosphate (r = −0.182, *p* < 0.001), PTH (r = −0.285, *p* < 0.001), serum albumin (r = 0.195, *p* < 0.001), and markers of kidney function such as eGFR (r = 0.266, *p* < 0.001) and uPCR (r = −0.133, *p* = 0.024) (Table 2). SOD activity varied significantly across CKD stage categories (*p* < 0.001), as determined by the Kruskal–Wallis test (Figure 2). Multivariate regression analysis showed that eGFR was the sole, strong, and independent predictor of SOD activity (B = 0.012, 95% CI 0.004 to 0.019, *p* = 0.002) (Table 3), although PTH, PWV, Hb, hydrogen phosphate, serum albumin, and proteinuria were evaluated as independent predictors but were excluded.

#### 3.1.6. CAT

CAT activity had a median value of 0.47 (0.09–0.82) cat/L, correlating with various hemodynamic central indices, including SBP (r = 0.343, *p* = 0.001), mean BP (r = 0.387, *p* < 0.001), DBP (r = 0.344, *p* = 0.001), central and peripheral cSBP and cDBP (r = 0.369, *p* < 0.001 and r = 0.345, *p* = 0.001, respectively), cPP (r = 0.211, *p* = 0.048), and pPP (r = 0.251, *p* = 0.014), as well as eGFR (r = 0.223, *p* = 0.029) (Table 2). In multiple regression analysis, cSBP was the sole independent predictor of CAT levels (B = 0.002, 95% CI 0.001 to 0.003, *p* < 0.001) (Table 3), while cDBP, cPP, and eGFR were evaluated as independent predictors but were excluded (Table 3).

### 3.2. Sensitivity Analysis

Sensitivity analyses were performed within the HD and PD subgroups to assess the independent contribution of clinical and biochemical variables to OS markers while accounting for potential confounders. Among HD patients, the duration of HD modality was independently associated with MDA levels (B = 0.179, 95% CI 0.116 to 0.243, *p* < 0.001) and remained significant after adjustment for TRG, presence of plaque, and hypertension (B = 0.172, 95% CI 0.127 to 0.216, *p* < 0.001), whereas time on PD, potassium, total cholesterol, TRG, LDL, hypertension, angina, presence of plaque, and PET test results did not independently predict MDA levels. PTH was the sole independent predictor of AOPP levels (B = −0.046, 95% CI −0.082 to −0.010, *p* = 0.012). Among dialysis patients, the PET test result was a strong independent predictor of oxALB levels (B = 0.592, 95% CI 0.287 to 0.897, *p* < 0.001), whereas none of the potential confounders evaluated—eGFR, HR, time on HD, time on PD, serum albumin, HDL, IMT, PET test, and stroke—significantly improved the predictive model for SOD levels. Finally, HD was independently associated with XO activity (B = −1.543, 95% CI −2.281 to −0.805, *p* < 0.001), while other potential confounders (PWV, total cholesterol, LDL, hydrogen phosphate, PTH, time on HD, and serum albumin) did not significantly improve the model’s predictive performance for XO activity.

## 4. Discussion

OS in CKD occurs very early, is gradually augmented in parallel with disease progression, and predisposes individuals to all-cause and cardiovascular mortality as well as progression to end-stage kidney disease (ESKD) [31]. In dialysis patients, OS is further increased and the dialysis modality seems to have a crucial role in its pathogenesis. In HD, the main culprit of OS is the bioincompatibility of dialysate, membranes, plastic tubes, connections and central venous catheters. The overproduction of free radicals is aggravated by anemia and the presence of chronic microinflammation [32]. Moreover, patients undergoing HD appear to have impaired antioxidant defense mechanisms, contributing to the worsening redox state [33]. A single session of HD has been shown to significantly reduce total antioxidant capacity (TAC), a marker reflecting the combined activity of endogenous and exogenous antioxidants [34]. On the other hand, in PD, patients may carry a higher OS burden compared to pre-dialysis CKD stage 3–4 patients, although this is lower than in HD patients. In PD, OS is triggered mainly by the non-physiologic composition of conventional PD solutions (high glucose concentration, increased osmolarity and acidic pH), which exerts both local and systemic effects via advanced glycation end products (AGEs) [35]. AGEs accumulate in the peritoneum, and this pathological deposition is associated with immature angiogenesis and peritoneal dysfunction, as reflected in changes in peritoneal equilibration test (PET test) results [36]. Serum AGE concentrations are inversely related to residual kidney function, and tissue AGE accumulation has been proposed as an early predictor of all-cause or cardiovascular death, potentially through the acceleration of arterial stiffness [37].

Among the multitude of OS biomarkers, we selected markers of lipid and protein oxidation, pro-oxidant enzyme activity, and antioxidant enzyme activity that have been investigated in relation to kidney function and vascular alterations, including arterial stiffness and vascular calcification, in patients with CKD, which may in turn be associated with clinically relevant outcomes.

Regarding the enzymes studied (CAT, SOD, and XO), we assessed their overall circulating enzymatic activities and examined their associations with clinically relevant parameters. Although these enzymes are predominantly intracellular, their circulating activities have been assessed in clinical studies, including in patients with CKD, as a readily measurable component of the systemic redox environment [38,39,40,41]. The circulating redox state reflects the combined contributions of endogenous and exogenous antioxidant systems and may provide clinically relevant information regarding the overall systemic oxidative and antioxidant balance. Nevertheless, circulating enzymatic activity should not be considered a direct surrogate for intracellular enzyme regulation or activity. The functional enzymatic capacity is the result of various distinct biological processes that do not necessarily change in parallel in the uremic environment. Alterations in gene expression or transcription, mRNA translation, protein abundance, and post-translational modifications may differentially affect the expression and activity of these enzymes. Therefore, measurements at the cellular or molecular level provide valuable, but necessarily partial, insight into their regulation and functional state. In the present study, our aim was to assess the overall circulating enzymatic activity and investigate its association with clinically relevant parameters in patients with CKD, while acknowledging that this approach does not directly capture intracellular processes.

MDA is an established marker of lipid peroxidation and has been associated with early atherogenesis and increased CAC [42,43]. At the molecular level, the aldehyde groups of MDA react with the terminal lysine residue amino groups of apoB-100, leading to the generation of MDA-modified LDL. These MDA-modified LDL particles are scavenged by macrophages in the vascular wall, causing primary pre-atherosclerotic damage [44]. At the same time, the accumulation of MDA-modified LDL causes endotheliocyte damage and endothelial dysfunction, resulting in increased vascular stiffness and loss of the ability to regulate vascular smooth muscle tone in response to shear stress [45]. In non-dialysis-dependent patients at different CKD stages, the levels of MDA were significantly higher compared with healthy controls and exhibited an increasing trend at worse CKD stages [9,46,47]. In HD patients, plasma levels of MDA were increased by 1.7 times in comparison with healthy individuals [48], and after an HD session, MDA concentration was significantly higher compared with the pre-dialysis levels [49]. These data show a close association of MDA levels with kidney function.

In our study, kidney function was a strong and independent predictor of MDA levels. The unexpected positive correlation between eGFR and MDA suggests that in real-world clinical studies, lipid peroxidation does not progress in a strict, linear fashion alongside progressive kidney function decline. Instead, MDA exhibited a non-linear trajectory in our cohort, a pattern similarly observed in a recent study of 143 diabetic patients, where MDA levels fluctuated and dipped across progressive early CKD stages rather than increasing linearly [50]. Because MDA is a highly specific byproduct of lipid peroxidation, we hypothesize that its fluctuations are heavily driven by our cohort’s underlying lipid profile (see Supplementary Figure S1), which shows parallel distribution patterns between MDA, LDL, and total cholesterol. The elevation of MDA observed in our dialysis groups likely reflects the worsening of dyslipidemia associated with renal replacement therapies. This is especially true for PD, where the high glucose content of the dialysis fluids exacerbates dyslipidemia.

Clinical data suggest a strong association between MDA-modified LDL and acute coronary syndromes (ACS), and its assessment has been proposed as an indicator of ACS severity [51]. In HD patients, MDA concentrations were correlated with coronary artery calcification (CAC—calculated by multirow spiral computed tomography), where patients with the highest MDA levels were four times more likely to have severe CAC [43] and prevalent CVD [52]. This observation is in line with our finding that both carotid atherosclerosis and established CVD were strongly associated with circulating MDA. When HD patients were divided into 5 groups according to the severity of CAC score, a strong positive correlation was revealed with MDA levels [53] and a high ratio of MDA-modified LDL/LDL has been suggested as an additional risk factor for CAC [54]. In our study, MDA levels were significantly associated with established CVD (particularly MI and angina) and presence of atherosclerosis (assessed by both plaque and cIMT). Interestingly, the presence of an atheromatous carotid plaque was an independent predictor of MDA levels, irrespective of the stage of kidney dysfunction.

The effect of OS on protein modification and structural alteration can be reflected in the amount of AOPPs and oxALB. AOPPs are generated after the oxidation of plasma proteins with chlorinated oxidants, such as chloramines and hypochlorous acid [55], and oxALB arises from the post-translational oxidation and glycation of human serum albumin [56]. Declining kidney function has been associated with an increase in AOPP levels [57], and the assessment of circulating AOPPs may predict the onset and progression of kidney disease [16]. It has been suggested that high levels of AOPPs could accurately identify patients with CKD stage 4–5 in an adult population with and without diabetes mellitus type 2 [58]. In the present study, AOPP levels varied significantly among subjects with different stages of CKD, with the highest levels observed in patients at stages 3 and 4. Moreover, AOPPs were significantly associated with both eGFR and proteinuria—different markers of kidney damage. This finding may reflect the increased oxidative burden associated with advanced CKD, even before the initiation of RRT. The assessment of AOPPs may be of particular interest in CKD, as their levels have been associated with PWV [59] and proposed as independent risk factors for CVD in predialysis patients [60]. Furthermore, in HD patients, higher serum AOPP levels contribute to sarcopenia [61] and exhibit a positive relationship with all-cause mortality [62]. Taken together, we can conclude that our findings show that the oxidation of proteins is associated with kidney damage and arterial stiffness in CKD.

In CKD, the kidneys’ inability to excrete excess hydrogen phosphate leads to a positive hydrogen phosphate balance and progressively to the development of mineral bone disease (MBD). The elevated levels of PTH and secondary hyperparathyroidism serve as compensatory mechanisms that are insufficient to counteract the disturbances in the calcium–hydrogen phosphate product [63]. Apart from the increased fracture risk, these biochemical abnormalities in mineral metabolism favor vascular and valvular calcification as well as left ventricular hypertrophy. According to the latest KDIGO guidelines on MBD, PTH must be assessed in relation to calcium and hydrogen phosphate [64], as a high calcium/hydrogen phosphate ratio can predict ACS [65] and serve as early atherosclerosis indicators in CKD patients [66]. Investigating the association between PTH and OS biomarkers, we found that in patients undergoing dialysis, PTH was a strong independent predictor of AOPP levels, even after adjustment for other cardiovascular, metabolic and renal confounders. In contrast, in a cross-sectional study that included 132 HD patients, Jaqueto et al. found no significant correlation between PTH and various OS biomarkers (TAC, MDA, lipid hydroperoxidation, AOPPs, nitric oxide metabolites, and 8-isoprostane) in HD patients [67], while there are reports of a reduction in OS (AOPPs and oxALB) after parathyroidectomy in patients with primary hyperparathyroidism [68]. Τhe impact of hyperparathyroidism on OS and, more specifically, on AOPP concentrations remains insufficiently described.

The correlation between OS and vascular dysfunction has been extensively studied [69]. In a healthy population without known CVD risk factors or markers of inflammation, the extracellular oxidant burden measured by cystine levels was associated with increased arterial stiffness and altered wave reflections [70]. Also, oxidative modifications of vasodilatory mediators and lipoproteins have been linked to alterations in peripheral arterial elastic properties [71]. Vascular elasticity is largely determined by the ratio of elastin to collagen in the vascular wall. Aging and atherosclerosis lead to reduced arterial compliance through elastic fiber degeneration and greater collagen fiber recruitment [72]. PWV can be used to determine arterial stiffness, and an increase in aortic PWV by just 1 m/s has been estimated to increase the risk of cardiovascular events by 14% [73]. Wykretowicz et al. found that AOPP concentration is elevated in otherwise healthy subjects with increased values of stiffness index [74]. In our study population, a similar association was revealed as well, as PWV, an established marker of arterial stiffness [75], was independently associated with the levels of oxALB and with XO activity, providing proof of the intrinsic link between redox status and vascular function in patients with CKD.

In kidney disease, reduced antioxidant capacity may result from increased consumption of antioxidants, decreased synthesis, uremia-related inhibition of enzyme activity, or even amino acid polymorphisms of antioxidant proteins [76,77]. SOD is a potent antioxidant enzyme, and its strong antioxidant activity is attributed to the ability to directly scavenge free radicals [78], as it catalyzes the dismutation reaction of the superoxide anion free radical to hydrogen peroxide and normal oxygen [79]. In 1996, Kashem et al. histologically examined cases of glomerulonephritis and suggested that low levels of SOD activity are related to reduced local antioxidant defense, oxidation of kidney cells and further kidney impairment [80]. Clinical studies have described a J-shaped pattern of circulating levels of SOD across CKD stages: SOD is progressively reduced in stages 1–4, possibly due to increasing OS and chronic inflammation, and then increases in ESKD through mechanisms that are not fully understood [81]. Conversely, Sagar et al. showed that serum SOD levels decline in parallel with the progression of CKD, with patients in CKD stage 5 having the lowest levels [82]. Based on our observations, SOD activity demonstrated significant variation among patients at different stages of kidney dysfunction. It remained relatively stable during the early stages of the disease, followed by a gradual decline with advancing CKD. SOD activity remained comparable between patients with stage 4 and those undergoing HD, whereas the lowest activity was observed among patients receiving PD. Among all markers of OS that were studied, the association of kidney function with SOD levels was by far the most robust. However, in 60 PD patients, Chen et al. observed higher SOD levels compared with 80 HD patients [83], while red cell SOD activity was comparable between 18 patients undergoing continuous ambulatory PD and 20 HD patients [84]. The discrepancy between these findings may reflect differences in patient characteristics, dialysis vintage, nutritional status or methodological differences in the assessment of SOD activity. The identification of eGFR as an independent predictor of SOD activity further supports the close interplay between renal function and antioxidant capacity. However, the conflicting patterns of SOD activity across different stages of CKD warrant further investigation in larger longitudinal cohorts.

CAT, another effective free-radical scavenging enzyme, plays a prominent role in hydrogen peroxide (H_2_O_2_) reduction and in the renal anti-oxidative shield [85]. In preclinical studies, CAT deficiency has been linked to accelerated renal fibrosis and progressive kidney injury [86]. Conversely, in transgenic mice, the overexpression of CAT has been shown to prevent hypertension and progression of nephropathy [87]. CAT levels are significantly lower in CKD patients in comparison to control groups [88], and its activity markedly decreases with increasing CKD stage [89]. CAT glycation, an irreversible modification typically occurring under conditions of OS, contributes to metabolic disorders like diabetes mellitus and neurodegenerative conditions and has been linked with accelerated atherosclerosis, impaired vascular elasticity and cardiac fibrosis [90]. In rat models after 5/6 nephrectomy, CAT activity significantly decreased in renal and aortic extracts, and this finding was coupled with marked arterial hypertension and significant urinary nitric oxide metabolite excretion [91]. In healthy individuals, the cardio-ankle vascular index (CAVI) was used to assess arterial stiffness, and a negative correlation was revealed with CAT levels [92]. There are also reports of certain treatment regimens (e.g., Linagliptin in diabetes mellitus type 2 and sirolimus in renal transplantation) that concomitantly increase CAT activity and improve parameters of arterial stiffness [93,94]. While the role of CAT in the pathogenesis of cardiovascular dysfunction is the subject of ongoing research and the causal relationships underlying these multifactorial biological processes remain to be elucidated, our study revealed an interesting association, with the hemodynamic parameter cSBP emerging as a strong independent predictor of CAT activity in CKD patients. This finding adds further evidence to the potential link between arterial stiffness and OS, suggesting a close pathophysiological interplay.

Our study has certain strengths: the large sample size, the inclusion of all CKD stages and controls, the measurement of markers of both arterial stiffness/calcification and the assessment of a complete panel of both oxidants and antioxidants, along with the prospective design that will shed some light on the possible association of OS with clinically relevant hard endpoints.

Several limitations of the present study should be acknowledged. First, its observational design limits our ability to establish causal relationships. The associations observed should therefore be interpreted in the context of the complex and potentially bidirectional interplay between declining kidney function, OS, arterial stiffness, and cardiovascular burden. Second, despite the large overall cohort, the CKD population was heterogeneous with respect to medical history, medication regimens, CKD stage, comorbidities, and functional status, which may have contributed to variability in oxidative and antioxidant profiles.

Furthermore, some of the findings observed in the present study may differ from those reported in previous experimental studies or other clinical populations. Such discrepancies may reflect the inherent differences between experimental models and real-world clinical populations and highlight the need for careful interpretation when extrapolating findings across different settings. Finally, the lack of methodological standardization in the assessment of OS biomarkers, including differences in analytical methods, sample matrices, and reporting procedures, may limit the comparability of our findings with those reported in the literature.

With regard to the CVD frequency that we reported, we acknowledge that given the high frequency of comorbidities such as diabetes mellitus and arterial hypertension in our cohort, the true prevalence of CVD is likely underreported and could be substantially higher. From our perspective, to maintain methodological rigor and data integrity, we solely reported CVD cases that were clinically validated and officially documented in medical records, deliberately excluding unverified or suspected diagnoses.

However, the relatively large study population and the prospective component of the present study provide clinically relevant information regarding the potential prognostic value of OS markers for hard clinical outcomes, including cardiovascular events and mortality. Further prospective studies in independent CKD populations are needed to confirm these findings and further clarify the prognostic significance of OS biomarkers.

Although the findings presented herein are derived from a cross-sectional analysis of the cohort, we plan to prospectively follow the participants over a 24-month period. These longitudinal analyses may help further characterize the clinical significance of the associations observed at baseline and enable the assessment of hard clinical endpoints.

## 5. Conclusions

By studying a broad panel of OS biomarkers in a large cohort of patients across the spectrum of CKD, we identified associations with both traditional and non-traditional cardiovascular risk factors. While some of these associations have been previously reported, our study also revealed novel or bidirectional ones. The main finding of our study was that kidney function, arterial stiffness and CVD were independent predictors of all OS markers in this cohort. The complex interplay between OS and the manifestations of CVD in patients with CKD is of clinical importance, and a better understanding of this overall picture may prove clinically valuable.

## Figures and Tables

**Table 1:** Baseline patients’ characteristics.

Characteristic	Value
Age, median [min–max]	69 [21–94]
Gender, male (%)	62.8% (312/497)
BMI, (mean ± SD)	27.2 ± 5.03
Stage of CKD (KDIGO 2012 Criteria), n	477
Stage 1 and 2 (eGFR > 60 mL/min/1.73 m^2^)	45
Stage 3 (eGFR > 30 mL/min/1.73 m^2^)	78
Stage 4 (eGFR > 15 mL/min/1.73 m^2^)	60
Stage 5 (eGFR < 15 mL/min/1.73 m^2^):	294
—Hemodialysis	164
—Peritoneal dialysis	130
Healthy controls	20
Disease prevalence (%)	
CVD	49.7% (247/490)
—MI	19.2% (94/488)
—Stroke	8.6% (42/489)
—Angina	17.6% (86/490)
—Heart Failure	28.9% (141/488)
—PAD	23.3% (114/488)
Diabetes Mellitus	42.3% (210/490)
Hypertension	74.8% (372/490)
Dyslipidemia	52.3% (260/490)
Disease Duration (years)	
CVD	10 [0–31]
Diabetes Mellitus	20 [0–54]
Hypertension	15 [0–45]
Hemodynamic Parameters (mmHg)	
SBP	140 [72–190]
MAP	103 [52–154]
DBP	86 [36–134]

Abbreviations: BMI: body mass index; CKD: chronic kidney disease; CVD: cardiovascular disease; DBP: diastolic blood pressure; eGFR: estimated glomerular filtration rate; KDIGO: Kidney Disease: Improving Global Outcomes; MAP: mean arterial pressure; MI: myocardial infarction; PAD: peripheral artery disease; SBP: systolic blood pressure; SD: standard deviation.

**Table 2:** Correlations of oxidative stress markers with various variables.

Parameters	r	*p*
MDA		
eGFR	0.126	0.008
Age	−0.167	0.001
PWV	−0.008	0.880
Systolic BP	−0.016	0.771
Mean BP	−0.006	0.918
Diastolic BP	0.051	0.341
Duration of CVD (years)	−0.092	0.059
PTH	−0.037	0.449
Hemoglobin	0.136	0.005
Potassium	0.171	<0.001
Calcium	0.109	0.024
Hydrogen phosphate	−0.007	0.889
Total cholesterol	0.181	<0.001
LDL	0.161	0.001
Serum albumin	0.042	0.393
Carotid IMT	−0.122	0.024
AOPPs		
eGFR	0.096	0.045
Age	0.064	0.185
PWV	−0.067	0.212
Systolic BP	0.027	0.612
Mean BP	0.036	0.507
Diastolic BP	0.022	0.679
Duration of CVD (years)	0.046	0.350
Calcium	−0.060	0.220
PTH	−0.109	0.025
Hydrogen phosphate	0.082	0.091
Total cholesterol	−0.130	0.008
LDL	−0.108	0.026
Carotid IMT	−0.051	0.289
uPCR	−0.117	0.048
oxALB		
eGFR	−0.075	0.137
Age	0.012	0.811
PWV	−0.134	0.017
Systolic BP	−0.079	0.161
Mean BP	−0.093	0.100
Diastolic BP	−0.091	0.107
Duration of CVD (years)	−0.014	0.778
PTH	0.109	0.032
Calcium	−0.045	0.378
Hydrogen phosphate	0.018	0.722
Total cholesterol	−0.018	0.728
Serum albumin	−0.018	0.731
Carotid IMT	0.042	0.457
SOD		
eGFR	0.266	<0.001
Age	−0.045	0.355
PWV	0.109	0.042
Systolic BP	0.017	0.760
Mean BP	0.033	0.541
Diastolic BP	0.034	0.534
Duration of CVD (years)	−0.029	0.550
Hemoglobin	0.110	0.023
Urea	−0.295	<0.001
Sodium	0.217	<0.001
PTH	−0.285	<0.001
Calcium	0.072	0.140
Hydrogen phosphate	−0.182	<0.001
Total cholesterol	0.020	0.688
Serum albumin	0.195	<0.001
Carotid IMT	−0.023	0.673
uPCR	−0.133	0.024
CAT		
eGFR	0.221	0.029
Age	0.077	0.458
PWV	0.052	0.618
Systolic BP	0.343	0.001
Mean BP	0.387	<0.001
Diastolic BP	0.344	0.001
cSBP	0.369	<0.001
cDBP	0.345	0.001
cPP	0.211	0.048
pPP	0.251	0.014
Duration of CVD (years)	−0.112	0.278
PTH	−0.109	0.289
Calcium	0.039	0.703
Hydrogen phosphate	−0.182	0.077
Total cholesterol	0.077	0.453
Serum albumin	0.034	0.745
Carotid IMT	−0.093	0.478
XO		
eGFR	0.006	0.906
Age	−0.102	0.037
PWV	−0.121	0.026
Systolic BP	−0.073	0.183
Mean BP	−0.045	0.409
Diastolic BP	−0.007	0.904
pPP	−0.117	0.032
Duration of CVD (years)	−0.100	0.042
PTH	−0.008	0.870
Calcium	−0.038	0.435
Hydrogen phosphate	0.024	0.618
Total cholesterol	0.089	0.069
Serum albumin	−0.066	0.182
Carotid IMT	−0.079	0.150

Abbreviations: AOPPs: advanced oxidation protein products; BP: blood pressure; CAT: catalase; cDBP: central diastolic blood pressure; cPP: central pulse pressure; cSBP: central systolic blood pressure; CVD: cardiovascular disease; DBP: diastolic blood pressure; eGFR: estimated glomerular filtration rate; IMT: intima-media thickness; LDL: low-density lipoprotein; MDA: malondialdehyde; OxALB: oxidized albumin; *p*: *p*-value; pPP: peripheral pulse pressure; PTH: parathormone; PWV: pulse wave velocity; r: Spearman’s correlation coefficient; SBP: systolic blood pressure; SOD: superoxide dismutase; uPCR: urine protein-to-creatinine ratio; XO: xanthine oxidase.

**Table 3:** Regression analyses of oxidative stress markers.

Predictors	B	95% CI	*p*
MDA			
Atheromatous plaque status	−1.12	−1.85 to −0.39	0.003
eGFR	0.013	0.001 to 0.024	0.027
AOPPs			
PTH	−0.046	−0.075 to −0.016	0.003
Total cholesterol	−0.162	−0.280 to −0.045	0.007
OxALB			
PWV	−0.009	−0.017 to −0.001	0.031
SOD			
eGFR	0.012	0.004 to 0.019	0.002
CAT			
cSBP	0.002	0.001 to 0.003	<0.001
XO			
PWV	−0.228	−0.415 to −0.040	0.017

Predictors evaluated in the analyses were selected based on the findings of the initial correlation analysis. The specific variables included in the final models and those excluded during the analysis for each biomarker are as follows: MDA: Included predictors were atheromatous plaque status and eGFR; evaluated but excluded variables were sex, Hb, calcium, and CVD. AOPPs: Included predictors were PTH and total cholesterol; evaluated but excluded variables were eGFR, LDL, and proteinuria. oxALB: The included predictor was PWV; PTH was evaluated but excluded. SOD: The included predictor was eGFR; evaluated but excluded variables were PTH, PWV, Hb, hydrogen phosphate, serum albumin, and proteinuria. CAT: The included predictor was cSBP; evaluated but excluded variables were cDBP, cPP, and eGFR. Abbreviations: 95% CI: 95% Confidence Interval; AOPPs: Advanced oxidation protein products; B: Unstandardized beta coefficient; CAT: Catalase; cDBP: Central diastolic blood pressure; cSBP: Central systolic blood pressure; CVD: Cardiovascular disease; eGFR: Estimated glomerular filtration rate; Hb: Hemoglobin; LDL: Low-density lipoprotein; MDA: Malondialdehyde; oxALB, Oxidized albumin; pPP: Peripheral pulse pressure; PTH: Parathormone; PWV: Pulse wave velocity; SOD: Superoxide dismutase; XO: Xanthine oxidase.

## Data Availability

The data presented in this study are available on request from the corresponding author due to ethical concerns.

## Supplementary Materials

The following supporting information can be downloaded at: https://www.mdpi.com/article/10.3390/antiox15101340/s1, Figure S1: Distribution of lipid parameters and malondialdehyde (MDA) across chronic kidney disease (CKD) stages and renal replacement therapies; Table S1: Patients’ Descriptive Statistics; Table S2: Correlations of oxidative stress markers with various variables after applying False Discovery Rate (FDR) procedure.

## Abbreviations

The following abbreviations are used in this manuscript:

ABPM	ambulatory blood pressure monitoring
ACS	acute coronary syndrome
AGEs	advanced glycation end products
AΙx	augmentation index
AKI	acute kidney injury
AOPPs	advanced oxidation protein products
BH	body height
BMI	body mass index
BP	blood pressure
BW	body weight
CAT	catalase
CAC	coronary artery calcification
CAVI	cardio-ankle vascular index
cDBP	central diastolic blood pressure
cIMT	carotid intima-media thickness
CKD	chronic kidney disease
CKD-EPI	Chronic Kidney Disease Epidemiology Collaboration
CRP	C-reactive protein
CVD	cardiovascular disease
cSBP	central systolic blood pressure
DBP	diastolic blood pressure
eGFR	estimated glomerular filtration rate
ESKD	end-stage kidney disease
FRD	false discovery rate
HbA1c	glycated hemoglobin
HD	hemodialysis
HDL	high-density lipoprotein
HR	heart rate
IMT	intima-media thickness
ISPD	International Society for Peritoneal Dialysis
KDIGO	Kidney Disease: Improving Global Outcomes
LDL	low-density lipoprotein
MAP	mean arterial pressure
MBD	mineral bone disease
MDA	malondialdehyde
MI	myocardial infarction
OS	oxidative stress
oxALB	oxidized albumin
PAD	peripheral artery disease
PD	peritoneal dialysis
PET	peritoneal equilibration test
PP	pulse pressure
pPP	peripheral pulse pressure
PTH	parathormone
PWV	pulse wave velocity
ROS	reactive oxygen species
RRT	renal replacement therapy
SBP	systolic blood pressure
SD	standard deviation
SOD	superoxide dismutase
TAC	total antioxidant capacity
TBARS	thiobarbituric acid reactive substances
TRG	triglycerides
uACR	urine albumin-to-creatinine ratio
uPCR	urine protein-to-creatinine ratio
XO	xanthine oxidase

## References

[1] Pizzino G., Irrera N., Cucinotta M., Pallio G., Mannino F., Arcoraci V., Squadrito F., Altavilla D., Bitto A. (2017). Oxidative Stress: Harms and Benefits for Human Health. Oxid. Med. Cell. Longev..

[2] Young I.S., Woodside J.V. (2001). Antioxidants in Health and Disease. J. Clin. Pathol..

[3] Krishnamurthy H.K., Pereira M., Rajavelu I., Jayaraman V., Krishna K., Wang T., Bei K., Rajasekaran J.J. (2024). Oxidative Stress: Fundamentals and Advances in Quantification Techniques. Front. Chem..

[4] Anwar S., Alharbi H.O.A., Babiker A.Y., Rahmani A.H. (2026). Oxidative Stress in Health and Disease: Mechanisms and Therapeutic Perspectives. Int. J. Mol. Sci..

[5] Sela S., Shurtz-Swirski R., Cohen-Mazor M., Mazor R., Chezar J., Shapiro G., Hassan K., Shkolnik G., Geron R., Kristal B. (2005). Primed peripheral polymorphonuclear leukocyte: A culprit underlying chronic low-grade inflammation and systemic oxidative stress in chronic kidney disease. J. Am. Soc. Nephrol..

[6] Chen M.F., Chang C.L., Liou S.Y. (1998). Increase in resting levels of superoxide anion in the whole blood of uremic patients on chronic hemodialysis. Blood Purif..

[7] Zhou Q., Wu S., Jiang J., Tian J., Chen J., Yu X., Chen P., Mei C., Xiong F., Shi W. (2012). Accumulation of circulating advanced oxidation protein products is an independent risk factor for ischaemic heart disease in maintenance haemodialysis patients. Nephrology.

[8] Stępniewska J., Dołęgowska B., Cecerska-Heryć E., Gołembiewska E., Malinowska-Jędraszczyk A., Marchelek-Myśliwiec M., Ciechanowski K. (2016). The activity of antioxidant enzymes in blood platelets in different types of renal replacement therapy: A cross-sectional study. Int. Urol. Nephrol..

[9] Tomás-Simó P., D’Marco L., Romero-Parra M., Tormos-Muñoz M.C., Sáez G., Torregrosa I., Estañ-Capell N., Miguel A., Gorriz J.L., Puchades M.J. (2021). Oxidative stress in non-dialysis-dependent chronic kidney disease patients. Int. J. Environ. Res. Public Health.

[10] Fonseca I., Reguengo H., Almeida M., Dias L., Martins L.S., Pedroso S., Santos J., Lobato L., Henriques A.C., Mendonça D. (2014). Oxidative stress in kidney transplantation: Malondialdehyde is an early predictive marker of graft dysfunction. Transplantation.

[11] Roumeliotis S., Roumeliotis A., Georgianos P.I., Stamou A., Manolopoulos V.G., Panagoutsos S., Liakopoulos V. (2021). Oxidized LDL Is Associated with eGFR Decline in Proteinuric Diabetic Kidney Disease: A Cohort Study. Oxid. Med. Cell. Longev..

[12] Roumeliotis A., Roumeliotis S., Tsetsos F., Georgitsi M., Georgianos P.I., Stamou A., Vasilakou A., Kotsa K., Tsekmekidou X., Paschou P. (2021). Oxidative Stress Genes in Diabetes Mellitus Type 2: Association with Diabetic Kidney Disease. Oxid. Med. Cell. Longev..

[13] Cottone S., Lorito M.C., Riccobene R., Nardi E., Mulè G., Buscemi S., Geraci C., Guarneri M., Arsena R., Cerasola G. (2008). Oxidative Stress, Inflammation and Cardiovascular Disease in Chronic Renal Failure. J. Nephrol..

[14] Duni A., Liakopoulos V., Roumeliotis S., Peschos D., Dounousi E. (2019). Oxidative Stress in the Pathogenesis and Evolution of Chronic Kidney Disease: Untangling Ariadne’s Thread. Int. J. Mol. Sci..

[15] Carracedo J., Alique M., Vida C., Bodega G., Ceprián N., Morales E., Praga M., De Sequera P., Ramírez R. (2020). Mechanisms of Cardiovascular Disorders in Patients With Chronic Kidney Disease: A Process Related to Accelerated Senescence. Front. Cell Dev. Biol..

[16] Conti G., Caccamo D., Siligato R., Gembillo G., Satta E., Pazzano D., Carucci N., Carella A., Campo G.D., Salvo A. (2019). Association of Higher Advanced Oxidation Protein Products (AOPPs) Levels in Patients with Diabetic and Hypertensive Nephropathy. Medicina.

[17] Soejima A., Matsuzawa N., Hayashi T., Kimura R., Ootsuka T., Fukuoka K., Yamada A., Nagasawa T., Era S. (2004). Alteration of Redox State of Human Serum Albumin before and after Hemodialysis. Blood Purif..

[18] Templar J., Kon S.P., Milligan T.P., Newman D.J., Raftery M.J. (1999). Increased plasma malondialdehyde levels in glomerular disease as determined by a fully validated HPLC method. Nephrol. Dial. Transplant..

[19] De Rojas A.H., Mateo M.C.M. (1996). Superoxide Dismutase and Catalase Activities in Patients Undergoing Hemodialysis and Continuous Ambulatory Peritoneal Dialysis. Ren. Fail..

[20] Terawaki H., Hayashi T., Murase T., Iijima R., Waki K., Tani Y., Nakamura T., Yoshimura K., Uchida S., Kazama J.J. (2018). Relationship between Xanthine Oxidoreductase Redox and Oxidative Stress among Chronic Kidney Disease Patients. Oxid. Med. Cell. Longev..

[21] Levin A., Stevens P.E. (2014). Summary of KDIGO 2012 CKD Guideline: Behind the Scenes, Need for Guidance, and a Framework for Moving Forward. Kidney Int..

[22] Morelle J., Stachowska-Pietka J., Öberg C., Gadola L., La Milia V., Yu Z., Lambie M., Mehrotra R., De Arteaga J., Davies S. (2021). ISPD Recommendations for the Evaluation of Peritoneal Membrane Dysfunction in Adults: Classification, Measurement, Interpretation and Rationale for Intervention. Perit. Dial. Int..

[23] Aguilar Diaz De Leon J., Borges C.R. (2020). Evaluation of Oxidative Stress in Biological Samples Using the Thiobarbituric Acid Reactive Substances Assay. JoVE (J. Vis. Exp.).

[24] Witko-Sarsat V., Friedlander M., Nguyen Khoa T., Capeillère-Blandin C., Nguyen A.T., Canteloup S., Dayer J.M., Jungers P., Drüeke T., Descamps-Latscha B. (1998). Advanced Oxidation Protein Products as Novel Mediators of Inflammation and Monocyte Activation in Chronic Renal Failure. J. Immunol..

[25] Bar–Or D., Lau E., Winkler J.V. (2000). A Novel Assay for Cobalt-Albumin Binding and Its Potential as a Marker for Myocardial Ischemia—A Preliminary Report. J. Emerg. Med..

[26] Prajda N., Weber G. (1975). Malignant Transformation-Linked Imbalance: Decreased Xanthine Oxidase Activity in Hepatomas. FEBS Lett..

[27] Góth L. (1991). A Simple Method for Determination of Serum Catalase Activity and Revision of Reference Range. Clin. Chim. Acta.

[28] Yang S., Jensen M.K., Rimm E.B., Willett W., Wu T. (2014). Erythrocyte Superoxide Dismutase, Glutathione Peroxidase, and Catalase Activities and Risk of Coronary Heart Disease in Generally Healthy Women: A Prospective Study. Am. J. Epidemiol..

[29] Roumeliotis A., Roumeliotis S., Panagoutsos S., Theodoridis M., Argyriou C., Tavridou A., Georgiadis G.S. (2019). Carotid Intima-Media Thickness Is an Independent Predictor of All-Cause Mortality and Cardiovascular Morbidity in Patients with Diabetes Mellitus Type 2 and Chronic Kidney Disease. Ren. Fail..

[30] Benjamini Y., Yekutieli D. (2001). The Control of the False Discovery Rate in Multiple Testing under Dependency. Ann. Stat..

[31] Roumeliotis S., Neofytou Ioannis E., Schurgers J., Liakopoulos V., Andreescu S., Henkel R., Khelfi A. (2024). Oxidative Stress in Chronic Kidney Disease. Biomarkers of Oxidative Stress.

[32] Liakopoulos V., Roumeliotis S., Gorny X., Dounousi E., Mertens P.R. (2017). Oxidative Stress in Hemodialysis Patients: A Review of the Literature. Oxid. Med. Cell. Longev..

[33] Morena M., Cristol J.-P., Canaud B. (2000). Why Hemodialysis Patients Are in a Prooxidant State? What Could Be Done to Correct the Pro/Antioxidant Imbalance. Blood Purif..

[34] Ogunleye A., Akinbodewa A.A., Adejumo O.A., Oluwafemi T.T., Akinfaderin D.A. (2018). Changes in Antioxidant Status Associated with Haemodialysis in Chronic Kidney Disease. Ghana Med. J..

[35] Liakopoulos V., Roumeliotis S., Gorny X., Eleftheriadis T., Mertens P.R. (2017). Oxidative Stress in Patients Undergoing Peritoneal Dialysis: A Current Review of the Literature. Oxid. Med. Cell. Longev..

[36] Nakano T., Mizumasa T., Kuroki Y., Eriguchi M., Yoshida H., Taniguchi M., Masutani K., Tsuruya K., Kitazono T. (2020). Advanced Glycation End Products Are Associated with Immature Angiogenesis and Peritoneal Dysfunction in Patients on Peritoneal Dialysis. Perit. Dial. Int..

[37] Steenbeke M., Speeckaert R., Desmedt S., Glorieux G., Delanghe J.R., Speeckaert M.M. (2022). The Role of Advanced Glycation End Products and Its Soluble Receptor in Kidney Diseases. Int. J. Mol. Sci..

[38] Vida C., Oliva C., Yuste C., Ceprián N., Caro P.J., Valera G., González De Pablos I., Morales E., Carracedo J. (2021). Oxidative Stress in Patients with Advanced CKD and Renal Replacement Therapy: The Key Role of Peripheral Blood Leukocytes. Antioxidants.

[39] Mimić-Oka J., Simić T., Djukanović L., Reljić Z., Davicević Z. (1999). Alteration in Plasma Antioxidant Capacity in Various Degrees of Chronic Renal Failure. Clin. Nephrol..

[40] Gondouin B., Jourde-Chiche N., Sallee M., Dou L., Cerini C., Loundou A., Morange S., Berland Y., Burtey S., Brunet P. (2015). Plasma Xanthine Oxidase Activity Is Predictive of Cardiovascular Disease in Patients with Chronic Kidney Disease, Independently of Uric Acid Levels. Nephron.

[41] Cecerska-Heryć E., Heryć R., Dutkiewicz G., Michalczyk A., Grygorcewicz B., Serwin N., Napiontek-Balińska S., Dołęgowska B. (2022). Xanthine Oxidoreductase Activity in Platelet-Poor and Rich Plasma as a Oxidative Stress Indicator in Patients Required Renal Replacement Therapy. BMC Nephrol..

[42] Cui X., Gong J., Han H., He L., Teng Y., Tetley T., Sinharay R., Chung K.F., Islam T., Gilliland F. (2018). Relationship between Free and Total Malondialdehyde, a Well-Established Marker of Oxidative Stress, in Various Types of Human Biospecimens. J. Thorac. Dis..

[43] Jung H.H., Choi D.H., Lee S.H. (2004). Serum Malondialdehyde and Coronary Artery Disease in Hemodialysis Patients. Am. J. Nephrol..

[44] Lankin V.Z., Tikhaze A.K., Kumskova E.M. (2012). Macrophages Actively Accumulate Malonyldialdehyde-Modified but Not Enzymatically Oxidized Low Density Lipoprotein. Mol. Cell. Biochem..

[45] Lankin V.Z., Tikhaze A.K., Melkumyants A.M. (2022). Malondialdehyde as an Important Key Factor of Molecular Mechanisms of Vascular Wall Damage under Heart Diseases Development. Int. J. Mol. Sci..

[46] Atamer A., Kocyigit Y., Ecder S.A., Selek S., Ilhan N., Ecder T., Atamer Y. (2008). Effect of Oxidative Stress on Antioxidant Enzyme Activities, Homocysteine and Lipoproteins in Chronic Kidney Disease. J. Nephrol..

[47] González Rico M., Puchades M.J., García Ramón R., Saez G., Tormos M.C., Miguel A. (2006). Effect of oxidative stress in patients with chronic renal failure. Nefrología.

[48] Lukoseviciene R., Ziaukiene G., Urbeliene J., Kalpokaite Z., Glemziene I. (2003). A study of oxidative stress markers and antioxidant system activity in the serum of hemodialysis patients. Medicina.

[49] Rao A.M., Apoorva R., Anand U., Anand C.V., Venu G. (2012). Effect of Hemodialysis on Plasma Myeloperoxidase Activity in End Stage Renal Disease Patients. Indian J. Clin. Biochem..

[50] Roumeliotis S., Neofytou I.E., Roumeliotis A., Veljkovic A., Cojic M., Kocic G. (2025). Association of Oxidative Stress Markers with Cardio-Kidney-Metabolic Parameters and Cardiovascular Disease in Patients with Type 2 Diabetes Mellitus. Biomolecules.

[51] Holvoet P. (1999). Malondialdehyde-Modified LDL as a Marker of Acute Coronary Syndromes. JAMA.

[52] Boaz M., Matas Z., Biro A., Katzir Z., Green M., Fainaru M., Smetana S. (1999). Serum Malondialdehyde and Prevalent Cardiovascular Disease in Hemodialysis. Kidney Int..

[53] Taki K., Takayama F., Tsuruta Y., Niwa T. (2006). Oxidative Stress, Advanced Glycation End Product, and Coronary Artery Calcification in Hemodialysis Patients. Kidney Int..

[54] Asamiya Y., Yajima A., Tsuruta Y., Otsubo S., Nitta K. (2013). Oxidised LDL/LDL-Cholesterol Ratio and Coronary Artery Calcification in Haemodialysis Patients. Nutr. Metab. Cardiovasc. Dis..

[55] Kalousová M., Zima T., Tesař V., Dusilová-Sulková S., Škrha J. (2005). Advanced Glycoxidation End Products in Chronic Diseases—Clinical Chemistry and Genetic Background. Mutat. Res. Fundam. Mol. Mech. Mutagen..

[56] Ling X.C., Kuo K.-L. (2018). Oxidative Stress in Chronic Kidney Disease. Ren. Replace. Ther..

[57] Sung C.-C., Hsu Y.-C., Chen C.-C., Lin Y.-F., Wu C.-C. (2013). Oxidative Stress and Nucleic Acid Oxidation in Patients with Chronic Kidney Disease. Oxid. Med. Cell. Longev..

[58] Villalpando-Sánchez D.C., Barajas-Medina C.A., Alvarez-Aguilar C., López-Ortiz G., Romero-Henríquez L.F., Gómez-García A. (2024). Advanced Oxidative Protein Products Had a Diagnostic Accuracy for Identifying Chronic Kidney Disease in Adult Population. Metabolites.

[59] Vinereanu I.-V., Peride I., Niculae A., Tiron A.T., Caragheorgheopol A., Manda D., Checherita I.A. (2021). The Relationship between Advanced Oxidation Protein Products, Vascular Calcifications and Arterial Stiffness in Predialysis Chronic Kidney Disease Patients. Medicina.

[60] Descamps-Latscha B., Witko-Sarsat V., Nguyen-Khoa T., Nguyen A.T., Gausson V., Mothu N., London G.M., Jungers P. (2005). Advanced Oxidation Protein Products as Risk Factors for Atherosclerotic Cardiovascular Events in Nondiabetic Predialysis Patients. Am. J. Kidney Dis..

[61] Kato H., Watanabe H., Imafuku T., Arimura N., Fujita I., Noguchi I., Tanaka S., Nakano T., Tokumaru K., Enoki Y. (2021). Advanced Oxidation Protein Products Contribute to Chronic Kidney Disease-Induced Muscle Atrophy by Inducing Oxidative Stress via CD36/NADPH Oxidase Pathway. J. Cachexia Sarcopenia Muscle.

[62] Zhou C., Zhang Y., Chen J., Mei C., Xiong F., Shi W., Zhou W., Liu X., Sun S., Tian J. (2021). Association between Serum Advanced Oxidation Protein Products and Mortality Risk in Maintenance Hemodialysis Patients. J. Transl. Med..

[63] Salera D., Merkel N., Bellasi A., De Borst M.H. (2025). Pathophysiology of Chronic Kidney Disease–Mineral Bone Disorder (CKD-MBD): From Adaptive to Maladaptive Mineral Homeostasis. Clin. Kidney J..

[64] Ketteler M., Evenepoel P., Holden R.M., Isakova T., Jørgensen H.S., Komaba H., Nickolas T.L., Sinha S., Vervloet M.G., Cheung M. (2025). Chronic Kidney Disease–Mineral and Bone Disorder: Conclusions from a Kidney Disease: Improving Global Outcomes (KDIGO) Controversies Conference. Kidney Int..

[65] Abass N.M., Yousef A.M., Sabet E.A., Kamal Y.M., El-Rashidy M.H. (2024). Study of Serum Calcium and Phosphorus Levels in Chronic Kidney Disease Patients with Acute Coronary Syndrome. Egypt. J. Intern. Med..

[66] Popovic P.S., Figurek A. (2025). Mineral-Bone Disorder in Chronic Kidney Diseases as Early Atherosclerosis Indicators. Endocr. Abstr..

[67] Jaqueto M., Delfino V.D.A., Bortolasci C.C., Barbosa D.S., Morimoto H.K., Frange R.F.N., Ferreira L.F.F., Guimarães F.B.D.S. (2016). Are PTH Levels Related to Oxidative Stress and Inflammation in Chronic Kidney Disease Patients on Hemodialysis?. J. Bras. Nefrol..

[68] Tanaka M., Tokunaga K., Maruyama T., Otagiri M., Tominaga Y., Itoh K., Matsushita K., Komaba H., Fukagawa M. (2011). Parathyroidectomy Markedly Reduces Oxidative Stress in a Patient with Primary Hyperparathyroidism. Ther. Apher. Dial..

[69] Guzik T.J., Touyz R.M. (2017). Oxidative Stress, Inflammation, and Vascular Aging in Hypertension. Hypertension.

[70] Patel R.S., Al Mheid I., Morris A.A., Ahmed Y., Kavtaradze N., Ali S., Dabhadkar K., Brigham K., Hooper W.C., Alexander R.W. (2011). Oxidative Stress Is Associated with Impaired Arterial Elasticity. Atherosclerosis.

[71] Kals J., Kampus P., Kals M., Zilmer K., Kullisaar T., Teesalu R., Pulges A., Zilmer M. (2006). Impact of Oxidative Stress on Arterial Elasticity in Patients with Atherosclerosis. Am. J. Hypertens..

[72] Anderson T.J. (2006). Arterial Stiffness or Endothelial Dysfunction as a Surrogate Marker of Vascular Risk. Can. J. Cardiol..

[73] Vlachopoulos C., Aznaouridis K., Stefanadis C. (2010). Prediction of Cardiovascular Events and All-Cause Mortality With Arterial Stiffness. J. Am. Coll. Cardiol..

[74] Wykretowicz A., Adamska K., Krauze T., Guzik P., Szczepanik A., Rutkowska A., Wysoki H. (2007). The Plasma Concentration of Advanced Oxidation Protein Products and Arterial Stiffness in Apparently Healthy Adults. Free Radic. Res..

[75] Pilz N., Heinz V., Ax T., Fesseler L., Patzak A., Bothe T.L. (2024). Pulse Wave Velocity: Methodology, Clinical Applications, and Interplay with Heart Rate Variability. Rev. Cardiovasc. Med..

[76] Podkowińska A., Formanowicz D. (2020). Chronic Kidney Disease as Oxidative Stress- and Inflammatory-Mediated Cardiovascular Disease. Antioxidants.

[77] Crawford A., Fassett R.G., Coombes J.S., Kunde D.A., Ahuja K.D.K., Robertson I.K., Ball M.J., Geraghty D.P. (2011). Glutathione Peroxidase, Superoxide Dismutase and Catalase Genotypes and Activities and the Progression of Chronic Kidney Disease. Nephrol. Dial. Transplant..

[78] Rosa A.C., Corsi D., Cavi N., Bruni N., Dosio F. (2021). Superoxide Dismutase Administration: A Review of Proposed Human Uses. Molecules.

[79] Brownlee M. (2001). Biochemistry and Molecular Cell Biology of Diabetic Complications. Nature.

[80] Kashem A., Endoh M., Yamauchi F., Yano N., Nomoto Y., Sakai H., Pronai L., Tanaka M., Nakazawa H. (1996). Superoxide Dismutase Activity in Human Glomerulonephritis. Am. J. Kidney Dis..

[81] Krueger K., Shen J., Maier A., Tepel M., Scholze A. (2016). Lower Superoxide Dismutase 2 (SOD2) Protein Content in Mononuclear Cells Is Associated with Better Survival in Patients with Hemodialysis Therapy. Oxid. Med. Cell. Longev..

[82] Sagar P., Shekhar R., Pranay K., Singh P.P., Kumar P. (2026). Oxidant and Antioxidant Levels in Different Stages of Chronic Kidney Disease. Cureus.

[83] Chen C.-H., Huang S.-C., Huang S.-W., Tsai S.-F., Huang Y.-C. (2024). Trace Elements Status and Their Associations With Related Antioxidant Enzyme Activities in Patients Receiving Peritoneal Dialysis and Hemodialysis. J. Ren. Nutr..

[84] Taylor J.E., Scott N., Bridges A., Henderson I.S., Stewart W.K., Belch J.J. (1992). Lipid Peroxidation and Antioxidants in Continuous Ambulatory Dialysis Patients. Perit. Dial. Int..

[85] Nandi A., Yan L.-J., Jana C.K., Das N. (2019). Role of Catalase in Oxidative Stress- and Age-Associated Degenerative Diseases. Oxid. Med. Cell. Longev..

[86] Kobayashi M., Sugiyama H., Wang D.-H., Toda N., Maeshima Y., Yamasaki Y., Masuoka N., Yamada M., Kira S., Makino H. (2005). Catalase Deficiency Renders Remnant Kidneys More Susceptible to Oxidant Tissue Injury and Renal Fibrosis in Mice. Kidney Int..

[87] Shi Y., Lo C.-S., Chenier I., Maachi H., Filep J.G., Ingelfinger J.R., Zhang S.-L., Chan J.S.D. (2013). Overexpression of Catalase Prevents Hypertension and Tubulointerstitial Fibrosis and Normalization of Renal Angiotensin-Converting Enzyme-2 Expression in Akita Mice. Am. J. Physiol. Ren. Physiol..

[88] Rasool M., Ashraf M.A.B., Malik A., Waquar S., Khan S.A., Qazi M.H., Ahmad W., Asif M., Khan S.U., Zaheer A. (2017). Comparative Study of Extrapolative Factors Linked with Oxidative Injury and Anti-Inflammatory Status in Chronic Kidney Disease Patients Experiencing Cardiovascular Distress. PLoS ONE.

[89] Tbahriti H.F., Kaddous A., Bouchenak M., Mekki K. (2013). Effect of Different Stages of Chronic Kidney Disease and Renal Replacement Therapies on Oxidant-Antioxidant Balance in Uremic Patients. Biochem. Res. Int..

[90] Alhumaydhi F.A., Younus H., Khan M.A. (2025). Catalase Functions and Glycation: Their Central Roles in Oxidative Stress, Metabolic Disorders, and Neurodegeneration. Catalysts.

[91] Sindhu R.K., Ehdaie A., Farmand F., Dhaliwal K.K., Nguyen T., Zhan C.-D., Roberts C.K., Vaziri N.D. (2005). Expression of Catalase and Glutathione Peroxidase in Renal Insufficiency. Biochim. Biophys. Acta BBA Mol. Cell Res..

[92] Chotimol P., Saehuan C., Kumphune S. (2016). Correlation between Cardio-Ankle Vascular Index and Biomarkers of Oxidative Stress. Scand. J. Clin. Lab. Invest..

[93] Awal H.B., Nandula S.R., Domingues C.C., Dore F.J., Kundu N., Brichacek B., Fakhri M., Elzarki A., Ahmadi N., Safai S. (2020). Linagliptin, When Compared to Placebo, Improves CD34+ve Endothelial Progenitor Cells in Type 2 Diabetes Subjects with Chronic Kidney Disease Taking Metformin and/or Insulin: A Randomized Controlled Trial. Cardiovasc. Diabetol..

[94] Joannidès R., Monteil C., De Ligny B.H., Westeel P.F., Iacob M., Thervet E., Barbier S., Bellien J., Lebranchu Y., Seguin S.G. (2011). Immunosuppressant Regimen Based on Sirolimus Decreases Aortic Stiffness in Renal Transplant Recipients in Comparison to Cyclosporine. Am. J. Transplant..

