# Supplementary material for: The Association of Kidney Function and Cardiovascular Disease Indices with Pro-Oxidant and Antioxidant Markers in CKD
Source: Antioxidants (Basel). 2026 Oct 16;15(10):1340. doi: 10.3390/antiox15101340 (PMC13701710; doi:10.3390/antiox15101340)
Supplement: Supplementary file 1 [file antioxidants-15-01340-s001.zip › antioxidants-4555689-supplementary.pdf]

Supplementary Materials:

Supplementary Table S1. Patients' Descriptive Statistics

Characteristic	Value
**Dialysis Parameters**	
Time on HD (months)	0 [0–289]
Time on PD (months)	0 [0–135]
Total Time on Dialysis (months)	0 [0–360]
**Hemodynamic Parameters**	
SBP (mmHg)	130 [68–197]
Mean BP (mmHg)	103 [52–161]
DBP (mmHg)	80 [36–134]
pPP (mmHg)	49 [16–101]
cSBP (mmHg)	118 [63–181]
cDBP (mmHg)	81 [36–137]
cPP (mmHg)	37 [16–85]
HR (bpm)	70 [53–127]
PWV (m/s)	8.8 [3.1–23]
AIx (%)	24 [5.0–50.0]
AIx75 (Standardized for 75 bpm) (%)	23.0 [6.0–70.0]
cIMT average (mm)	7.83 [4.17–16]
**Renal Function Markers**	
eGFR (mL/min/1.73m^2)	9 [0–140]
Serum Creatinine (mg/dL)	4.34 [0.57–18.9]
Urea (mg/dL)	89 [16–240]

Characteristic	Value
Proteinuria (g/24h)	0.271 [0.030–33.000]
Albuminuria (g/24h)	0.180 [0.006–19.300]
**Serum Biochemistry & Electrolytes**	
Hemoglobin (g/dL)	12 [7.2–18.7]
HbA1c (%)	5.8 [3–10.7]
Serum Albumin (g/dL)	4 [2.1–6.50]
Calcium (mg/dL)	9.10 [6.17–11.30]
Phosphate (mg/dL)	4.2 [1.70–11]
Potassium (mEq/L)	4.60 [2.90–6.90]
Sodium (mEq/L)	138 [123–148]
PTH (pg/mL)	146 [5–2129]
CRP (mg/dL)	0.53 [0.05–77]
**Lipid Profile**	
Total Cholesterol (mg/dL)	157 [41–391]
Triglycerides (mg/dL)	133 [17–879]
HDL Cholesterol (mg/dL)	43 [3–139]
LDL Cholesterol (mg/dL)	81 [5–232]
**Oxidative Stress & Novel Biomarkers**	
MDA (μM/L)	3.56 [1.64–25.71]
AOPPs (mmol/L chloramine-T)	68.18 [0.17-239.43]
Ox ALB (Absorbance Units)	0.008 [0–0.85]
SOD activity (U/mL)	8.55 [0.53–9.68]
CAT (cat/L)	0.470 [0.094–0.82]
XO (mU/L)	1.37 [0.03–41.28]

Abbreviations: AIx: augmentation index; AIx75: augmentation index standardized for 75 bpm; AOPPs: advanced oxidation protein products; BP: blood pressure; bpm: beats per minute; CAT: catalase; cDBP: central diastolic blood pressure; cIMT: carotid intima-media thickness; cPP: central pulse pressure; CRP: C-reactive protein; cSBP: central systolic blood pressure; DBP: diastolic blood pressure; eGFR: estimated glomerular filtration rate; HbA1c: glycated hemoglobin; HD: hemodialysis; HDL: high-density lipoprotein; HR: heart rate; LDL: low-density lipoprotein; MDA: malondialdehyde; Ox ALB: oxidized albumin; PD: peritoneal dialysis; pPP: peripheral pulse pressure; PTH: parathyroid hormone; PWV: pulse wave velocity; SBP: systolic blood pressure; SOD: superoxide dismutase; XO: xanthine oxidase.

Supplementary Table S2. Correlations of oxidative stress markers with various variables after applying False Discovery Rate (FDR) procedure.

Parameters	r	p	FDR adj. p-value
	**MDA**		
**eGFR**	**0.126**	**0.008**	**0.040**
**Age**	**-0.167**	**0.001**	**0.006**
PWV	-0.008	0.880	0.916
Systolic BP	-0.016	0.771	0.843
Mean BP	-0.006	0.918	0.918
Diastolic BP	0.051	0.341	0.564
Duration of CVD (years)	-0.092	0.059	0.145
PTH	-0.037	0.449	0.641
**Hemoglobin**	**0.136**	**0.005**	**0.028**
**Potassium**	**0.171**	**<0.001**	**<0.001**
Calcium	0.109	0.024	0.088
Phosphorus	-0.007	0.889	0.916
**Total cholesterol**	**0.181**	**<0.001**	**<0.001**
**LDL**	**0.161**	**0.001**	**0.006**
Serum albumin	0.042	0.393	0.606
Carotid IMT	-0.122	0.024	0.088
	**AOPPs**		
eGFR	0.096	0.045	0.120
Age	0.064	0.185	0.344

PWV	-0.067	0.212	0.386
Systolic BP	0.027	0.612	0.770
Mean BP	0.036	0.507	0.689
Diastolic BP	0.022	0.679	0.824
Duration of CVD (years)	0.046	0.350	0.567
Calcium	-0.060	0.220	0.393
PTH	-0.109	0.025	0.088
Phosphorus	0.082	0.091	0.207
Total cholesterol	-0.130	0.008	0.040
LDL	-0.108	0.026	0.088
Carotid IMT	-0.051	0.289	0.487
uPCR	-0.117	0.048	0.121
**oxALB**			
eGFR	-0.075	0.137	0.290
Age	0.012	0.811	0.868
PWV	-0.134	0.017	0.077
Systolic BP	-0.079	0.161	0.319
Mean BP	-0.093	0.100	0.222
Diastolic BP	-0.091	0.107	0.232
Duration of CVD (years)	-0.014	0.778	0.843
PTH	0.109	0.032	0.097
Calcium	-0.045	0.378	0.593
Phosphorus	0.018	0.722	0.832
Total cholesterol	-0.018	0.728	0.832
Serum albumin	-0.018	0.731	0.832
Carotid IMT	0.042	0.457	0.641
**SOD**			
Age	-0.045	0.355	0.567
**eGFR**	**0.266**	**<0.001**	**<0.001**
PWV	0.109	0.042	0.116
Systolic BP	0.017	0.760	0.843

Mean BP	0.033	0.541	0.713
Diastolic BP	0.034	0.534	0.713
Duration of CVD (years)	-0.029	0.550	0.715
Hemoglobin	0.110	0.023	0.088
**Urea**	**-0.295**	**<0.001**	**<0.001**
**Sodium**	**0.217**	**<0.001**	**<0.001**
**PTH**	**-0.285**	**<0.001**	**<0.001**
Calcium	0.072	0.140	0.290
Phosphorus	-0.182	<0.001	<0.001
Total cholesterol	0.020	0.688	0.824
**Serum albumin**	**0.195**	**<0.001**	**<0.001**
Carotid IMT	-0.023	0.673	0.824
uPCR	-0.133	0.024	0.088
	**CAT**		
eGFR	0.221	0.029	0.094
Age	0.077	0.458	0.641
PWV	0.052	0.618	0.770
**Systolic BP**	**0.343**	**0.001**	**0.006**
**Mean BP**	**0.387**	**<0.001**	**<0.001**
**Diastolic BP**	**0.344**	**0.001**	**0.006**
**cSBP**	**0.369**	**<0.001**	**<0.001**
**cDBP**	**0.345**	**0.001**	**0.006**
cPP	0.211	0.048	0.121
pPP	0.251	0.014	0.067
Duration of CVD (years)	-0.112	0.278	0.487
PTH	-0.109	0.289	0.487
Calcium	0.039	0.703	0.831
Phosphorus	-0.182	0.077	0.180
Total cholesterol	0.077	0.453	0.641
Serum albumin	0.034	0.745	0.837
Carotid IMT	-0.093	0.478	0.659

**XO**			
eGFR	0.006	0.906	0.916
Age	-0.102	0.037	0.109
PWV	-0.121	0.026	0.088
Systolic BP	-0.073	0.183	0.344
Mean BP	-0.045	0.409	0.620
Diastolic BP	-0.007	0.904	0.916
pPP	-0.117	0.032	0.097
Duration of CVD (years)	-0.100	0.042	0.116
PTH	-0.008	0.870	0.916
Calcium	-0.038	0.435	0.641
Phosphorus	0.024	0.618	0.770
Total cholesterol	0.089	0.069	0.165
Serum albumin	-0.066	0.182	0.344
Carotid IMT	-0.079	0.150	0.303

FDR-Adj. p value: Adjusted p-values calculated using the Benjamini-Hochberg False Discovery Rate (FDR) procedure to correct for the large number of bivariate tests applied, effectively controlling the proportion of false positives.
Abbreviations: AOPPs: advanced oxidation protein products; BP: blood pressure; CAT: catalase; cDBP: central diastolic blood pressure; cPP: central pulse pressure; cSBP: central systolic blood pressure; CVD: cardiovascular disease; DBP: diastolic blood pressure; eGFR: estimated glomerular filtration rate; IMT: intima-media thickness; LDL: low-density lipoprotein; MDA: malondialdehyde; OxALB: oxidized albumin; p: p-value; pPP: peripheral pulse pressure; PTH: parathyroid hormone; PWV: pulse wave velocity; r: Spearman's correlation coefficient; SBP: systolic blood pressure; SOD: superoxide dismutase; uPCR: urine protein-to-creatinine ratio; XO: xanthine oxidase.

Supplementary Figure S1. Distribution of lipid parameters and malondialdehyde (MDA) across chronic kidney disease (CKD) stages and renal replacement therapies.

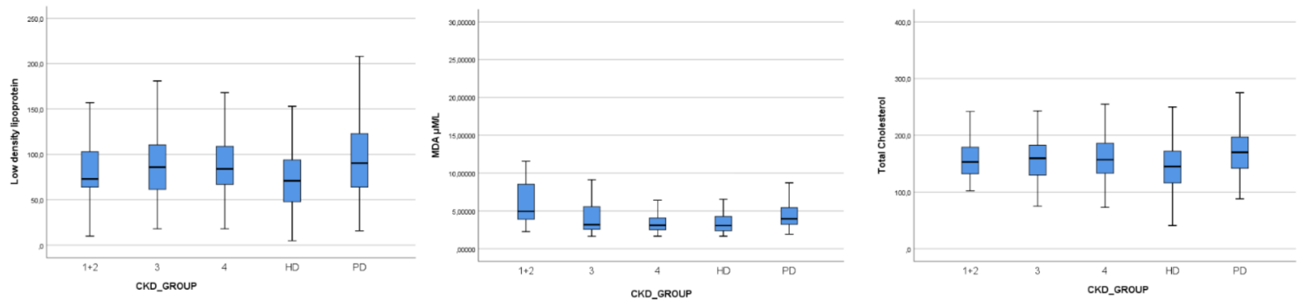

The boxplots demonstrate visually parallel distribution patterns among Low-Density Lipoprotein (LDL), MDA, and Total Cholesterol across CKD stages 1+2, 3, 4, hemodialysis (HD), and peritoneal dialysis (PD) groups.
